# An intron-split microRNA mediates cleavage of the mRNA encoded by low phosphate root in Solanaceae

**DOI:** 10.1007/s00425-024-04596-8

**Published:** 2025-01-07

**Authors:** Zahara Medina-Calzada, Runchun Jing, Simon Moxon, Hong Zhu, Ping Xu, Tamas Dalmay

**Affiliations:** 1 School of Biological Sciences, University of East Anglia, Norwich Research Park, Norwich, UK; 2 South China Botanical Garden, Chinese Academy of Sciences, Guangzhou, 510650 China; 3 Present Address: Shanghai Engineering Research Center of Plant Germplasm Resource, College of Life Sciences, Shanghai Normal University, Shanghai, 200234 China

**Keywords:** Ferroxidase LPR, MiRNA, MiRNA biogenesis, MiRNA conservation, Phosphate starvation, *Solanum*

## Abstract

**Main conclusion:**

A microRNA with a non-canonical precursor structure harbours an intron in between its miRNA-5p and miRNA-3p relevant for its biogenesis, is conserved across Solanaceae, and targets the mRNA of low phosphate root.

**Abstract:**

Hundreds of miRNAs have been identified in plants and great advances have been accomplished in the understanding of plant miRNA biogenesis, mechanisms and functions. Still, many miRNAs, particularly those with less conventional features, remain to be discovered. Likewise, additional layers of regulation from miRNA generation to action and turnover are still being revealed. The current study describes a microRNA not previously identified given its unusual intron-split stem-loop structure, that has been previously observed only within the monocot-specific miRNA444 family. It shows its conservation across a branch of Solanales including agriculturally relevant Solanaceae family, where its transcripts had already been predicted in several species within sequence databases. The miRNA is absent in *Arabidopsis thaliana* but present in *Solanum lycopersicum*, *Nicotiana benthamiana*, *Petunia axillaris*, and *Ipomoea nil.* It proves that at least two different pri-miRNA variants are produced from this miRNA gene, one spliced and the other one retaining the intron. It demonstrates the dual function of its intron in the miRNA biogenesis. On the one hand, its presence in the pri-miRNA positively influences mature miRNA accumulation, but on the other hand, it needs to be removed from the pri-miRNA for efficient mature miRNA production. Finally, it sets low phosphate root as one of its targets, a protein known to be involved in root growth regulation under phosphate starvation in other plant species.

**Supplementary Information:**

The online version contains supplementary material available at 10.1007/s00425-024-04596-8.

## Introduction

MicroRNAs (miRNAs) are small RNAs of 20–25 nucleotides in length generated by the precise excision of stem-loop structures folded within longer, single-stranded transcripts named primary miRNAs (pri-miRNA) (Ambros et al. 2003; Meyers et al. 2008). These hairpins are processed into miRNA duplexes by the endonuclease activity of the Dicer-like1 (DCL1) protein complex (Park et al. 2002; Reinhart et al. 2002; Kurihara and Watanabe 2004). Structural features of the pri-miRNA stem-loop direct its cleavage in a few possible patterns either from base to loop or in the opposite direction (Addo-Quaye et al. 2009; Bologna et al. 2009, 2013; Mateos et al. 2010; Song et al. 2010; Werner et al. 2010). After methylation at the 3′ end, mature miRNAs are loaded into the ARGONAUTE1 (AGO1) protein and exported to the cytoplasm to direct the cleavage or repress the translation of one or several complementary mRNA targets (Vaucheret et al. 2004; Baumberger and Baulcombe 2005; Bologna et al. 2018).

New miRNA genes (*MIRs*) have been proposed to arise from inverted duplications of coding genes that sometimes become their targets, from transposable elements such as miniature inverted-repeat transposable elements (MITEs) and from random genomic sequences which are either highly degenerated inverted duplications or self-complementary simply by chance (Allen et al. 2004; Fahlgren et al. 2007; De Felippes et al. 2008; Piriyapongsa and Jordan 2008; Roberts et al. 2014). Through time, new *MIRs* can expand into multigene miRNA families and can acquire new targets and expression patterns (Maher et al. 2006; Palatnik et al. 2007). There are *MIR* families conserved across all vascular plants, suggesting that they appeared early during the evolution of land plants. However, there are also *MIRs* that evolved later and therefore are not conserved in all plants. They tend to have a single copy with a specialised role within a specific lineage (Kutter et al. 2007; Cuperus et al. 2011).

Most times, plant *MIRs* appear scattered in the genome as independent intergenic transcriptional units that produce a single mature miRNA each (Rajagopalan et al. 2006; Griffiths-Jones et al. 2008). They show great variability in length and structure and frequently contain introns (Xie et al. 2005; Zhang et al. 2009; Szarzynska et al. 2009; Stepien et al. 2017). Many independent plant *MIRs* also show alternative splicing and alternative transcription start and polyadenylation sites (Xie et al. 2005; Hirsch et al. 2006; Song et al. 2007; Szarzynska et al. 2009; Bielewicz et al. 2013; Jia and Rock 2013; Kruszka et al. 2013; Schwab et al. 2013; Barciszewska-Pacak et al. 2016). Furthermore, it has been observed that the mere presence of introns in the pri-miRNA can enhance mature miRNA production (Bielewicz et al. 2013; Schwab et al. 2013).

In most cases, the miRNA hairpin is located in a single exon of the pri-miRNA (Szweykowska-Kulińska et al. 2013; Stepien et al. 2017). However, there are reports of *MIRs* where the miRNA stem-loop includes an exonic and an intronic part in alternatively spliced transcripts, so miRNA biogenesis is directly affected by alternative splicing, that itself is regulated by different stimulus (Hirsch et al. 2006; Jia and Rock 2013; Barciszewska-Pacak et al. 2016). Moreover, it has been found that members of the monocot-specific miRNA444 family harbour an intron in between miRNA-5p and miRNA-3p sequence (Sunkar et al. 2005; Lu et al. 2008). This peculiar intron–exon structure that was first observed in rice has been subsequently reported in other genes of the same family in maize, sorghum, and barley (Paterson et al. 2009; Zhang et al. 2009; Thieme et al. 2011; Chojnacka et al. 2023). It was even speculated that they could represent a class of intron-split miRNAs (Lu et al. 2008; Pek and Okamura 2015). However, to our knowledge, no other *MIRs* with this characteristic have been observed outside the miRNA444 family, even though there is a bioinformatic tool (SplamiR) specifically developed to identify this particular kind of pri-miRNAs (Thieme et al. 2011).

Here, we present an intron-split miRNA (miRtop14) conserved across Solanaceae that targets low phosphate root (LPR), a family of ferroxidases first described in *Arabidopsis* (Svistoonoff et al. 2007). These enzymes have been found to play a role in phosphate starvation signalling in root meristems, where they trigger iron and callose deposition in both *Arabidopsis* and rice (Svistoonoff et al. 2007; Müller et al. 2015; Cao et al. 2016; Ding et al. 2018). Beyond root, LPR mutations influence diverse morphological traits in rice (Ai et al. 2020). However, in spite of being widely distributed in the plant kingdom (Ming et al. 2013), their roles in other plants and tissues have not been studied yet.

## Materials and methods

### Plant materials and growth conditions

All plants were grown at 22 °C and 16 h light/ 8 h dark in a growth chamber. The species and cultivars/ecotypes used in this study were: *Solanum lycopersicum* cv. Ailsa Craig, *S. lycopersicum* cv. MicroTom, *Nicotiana benthamiana*, *Petunia axillaris* line S26, *Ipomoea nil* cv. Kikyo-zaki, *Arabidopsis thaliana* ecotype Columbia (Col-0), and *Oryza sativa* ssp. Japonica cv. Nipponbare.

### Database BLAST search and target prediction

*S. lycopersicum* pri-miRNA sequence with and without intron (uncharacterized LOC101267134, NCBI reference sequence XR_182935.5) was used to perform a BLAST search of nucleotide databases (Fernandez-Pozo et al. 2015; Hirakawa et al. 2015; Sayers et al. 2022). First, *Solanum* species were examined, and after the *MIR* was found in several species within the *Solanum* genus, the whole Solanaceae family was included in the search. Newly identified *MIRtop14* sequences were in turn used for BLAST searches against the next related species according to the Solanaceae phylogeny. After identifying the *MIR* gene in *Solanum*, *Capsicum*, *Nicotiana*, and *Petunia*, we expanded the search to the whole Solanales order. Using *Petunia axilaris MIR* sequence, we found *MIRtop14* within *Ipomoea* genus. Finally, we looked for *MIRtop14* sequence within the three closer orders to Solanales: Gentianales, Lamiales, and Boraginales (Refulio-Rodriguez and Olmstead 2014). MiRNA targets were predicted using the psRNAtarget software (Dai and Zhao 2011).

### Total RNA extractions, northern blot, RT-PCR and RLM-RACE analysis

Plant tissues were frozen and grinded in liquid nitrogen before RNA extraction was performed with Tri-reagent following the manufacturer’s protocol (Ambion). RNA was precipitated through addition of three volumes 100% ethanol, washed two times with 75% ethanol, and air dried before being dissolved in distilled water. The concentration and quality were measured using a NanoDrop spectrophotometer (Thermo Fisher Scientific) at an absorbance ratio of A260/280 and A260/230 nm. Two micrograms of total RNA from each sample were analysed by northern-blot analysis carried out as described by Pall et al. (2007). For northern-blot analysis across Solanales species and *A. thaliana,* the probe used was complementary to miRNAtop14 last 20 nucleotides, which are predicted to be the same in all four Solanales species analysed (only the first miRNAtop14 5′ end nucleotide changes between species). For northern-blot analysis from *S. lycopersicum* tissues and from GoldenGate constructs, the probe used was the full 21 nucleotides complementary to Sly-miRNAtop14. To detect Osa-miRNA528, the oligonucleotide 5′-CTCCTCTGCATGCCCCTTCCA-3′ was used. After stripping, an oligonucleotide complementary to U6 RNA (5′-AGGGGCCATGCTAATCTTCTC-3′) was used to demonstrate equal loading.

The same total RNA samples used for northern blot analysis were used for reverse-transcription and PCR analysis of pri-miRNAtop14. Detailed description of the RT-PCR analysis is described in https://ueaeprints.uea.ac.uk/id/eprint/67671/1/Zahara_Medina_Calzada_Thesis.pdf. Primers used are listed in Suppl. Table S1.

RLM-RACE analysis was carried out as described by Moxon et al. (2008). Primers used are listed in Suppl. Table S2.

### Cloning and sequencing

PCR products were run in agarose gel and bands recovered using the Zymoclean Gel DNA Recovery Kit, following manufacturer’s protocol (Zymo Research). PCR amplicons were ligated into pGEM-T Easy vectors according to the ligation protocol provided by the pGEM-T Easy Vector Systems manufacturer. The products of each reaction were cloned into *Escherichia coli* DH5α by heat-shock. Positive colonies were confirmed by sequencing (Eurofins MWG Operon, Ebersberg, Germany).

A scheme of the three GoldenGate constructs used in this study can be seen in Suppl. Fig. S1. The golden gate cloning method followed in this study was based in the golden gate system published by Weber et al. (2011)**.** Each assembly mixture contained 1 μL of vector backbone (100 ng), 1 μL of each additional assembly plasmid (100 ng), 1.5 μL of 10 × T4 ligase buffer, 0.15 μL 100X BSA, 1 μL T4 ligase (400,000 cohesive end units/ mL, New England Biolabs), 1 μL type II restriction enzyme (Bpi I for level 0 and level 2 assembly, Bsa I for level 1 assembly), and water up to 15 μL. The reaction consisted in 25 cycles of 3 min at 37 °C (restriction) followed by 4 min at 16 °C (ligation) and a final cycle of 5 min at 50 °C and 5 min at 80 °C. Level 0 and level 1 constructs positive colonies were checked by colony PCR and sequencing. Besides, level 2 constructs were checked by restriction digest additionally to specific modules sequencing, to confirm that the whole assembly was correct given the impossibility to amplify the large multi-gene fragment. For sequencing and restriction digest, plasmid DNA was isolated through miniprep using the QIAprep Spin Miniprep Kit. PCR amplification of *Osa-MIR528* sequence was carried out using primers 5′-tgaagacggaatgCCAGTGCACCATGGCCGG-3′ and 5′-tgaagacggaagcTTGTCGTTGACAATACTACTCTTCT-3′ and rice genomic DNA as a template. The recovered PCR products were assembled into a level 0 Golden Gate cloning vector. To create a non-spliceable intron containing *MIRtop14* construct, the 5’ splice site (SS) of the gene was mutated from G/GT to C/CC through site directed mutagenesis from the level 0 golden gate construct harbouring the WT *MIRtop14*. Primers were design to amplify the whole level 0 construct and two internal primers to introduce the mutations at the *MIRtop14* 5′SS: 5′-GTTTAATTTATTAC**CCC**ATGTTATTTGTC-3′ (this forward primer contains the 5′ splicing site sequence, but harbours a CCC sequence at this position instead of the original GGT) and 5′-AAACAATATTGATAAGCACTCTTT-3′ (it is adjacent to the forward primer, immediately upstream, but oriented towards the opposite direction).

### *Arabidopsis* transformation

The three level 2 constructs carried by *E. coli* DH5α bacteria were transferred into *Agrobacterium tumefaciens* GV3101 (pMP90) by electroporation using a MicroPulser Electroporation apparatus (Bio-Rad), following the instructions given by the manufacturer for the electroporation of *A. tumefaciens* and used to transform *Arabidopsis* plants by the floral dip method (Clough and Bent 1998). Three independent transgenic lines per construct were chosen for the analysis.

## Results

### The first intron-split miRNA found in *S. lycopersicum*

**Fig. 1:**
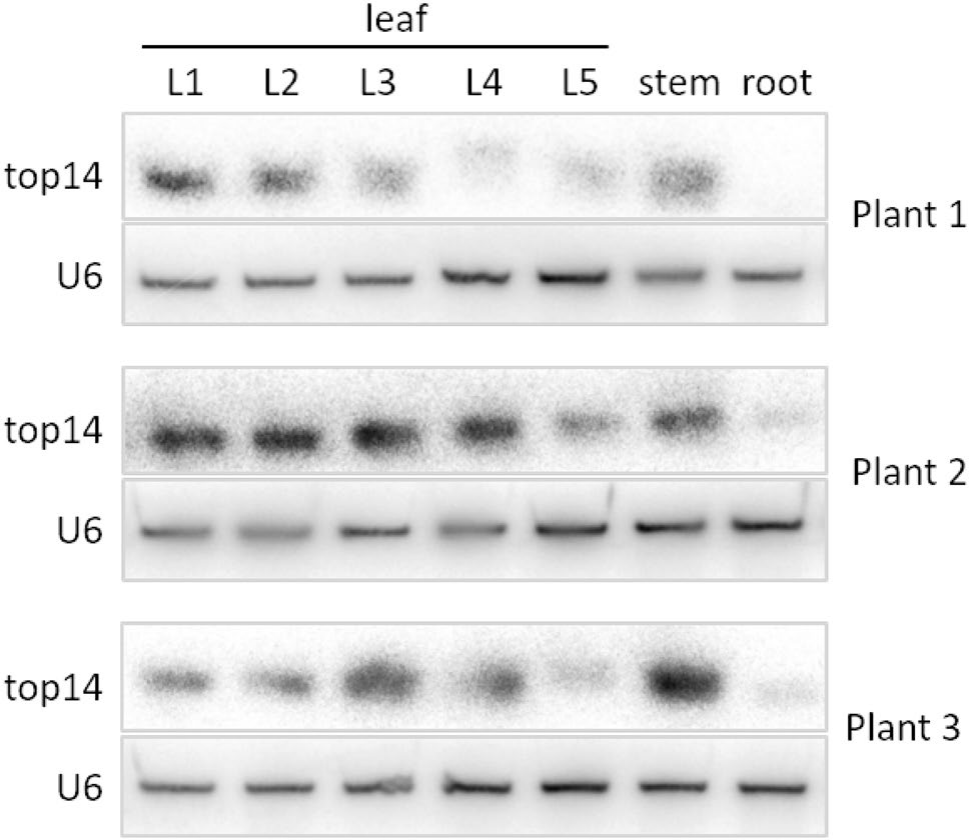
Detection of mature miRNAtop14 levels in *Solanum lycopersicum* root, stem, and leaves by northern blot. Different leaves from the same plant were named L1–L5, from the oldest to the youngest

We reported a deep-sequencing study of small RNAs from *S. lycopersicum* before the genome sequence was available (Moxon et al. 2008). Since we were not able to map the sequencing reads to the genome, we tried to find new miRNAs by looking for fully or partially reverse complementary sequence reads in the dataset, that would be generated from the two arms of pre-miRNAs (i.e., miRNA-5p and miRNA-3p). Initially, we focused on one particular read-pair, because one of the reads, the putative mature miRNA, was the 14th most abundant read in the dataset (therefore, we initially named it as putative miRNAtop14). We decided to test the presence of this putative miRNA in *S. lycopersicum* by northern blot, and confirmed its presence in stem, roots, and leaves of 1 week old plantlets (Fig. 1).

Later, when the *S. lycopersicum* genome became available, we aligned these two reads to the genome and found that the putative miRNA-5p and miRNA-3p were unusually far apart. The distance between them was 700 bp, while 98% of plant miRNA hairpins have a maximum length of slightly above 300 nucleotides (Thakur et al. 2011). This pri-miRNA would contain such a large loop in the miRNA hairpin that subsequent dicing was expected to be compromised. However, the mature miRNA was produced at clearly detectable levels in several tissues by northern blot analysis (Fig. 1).

**Fig. 2:**
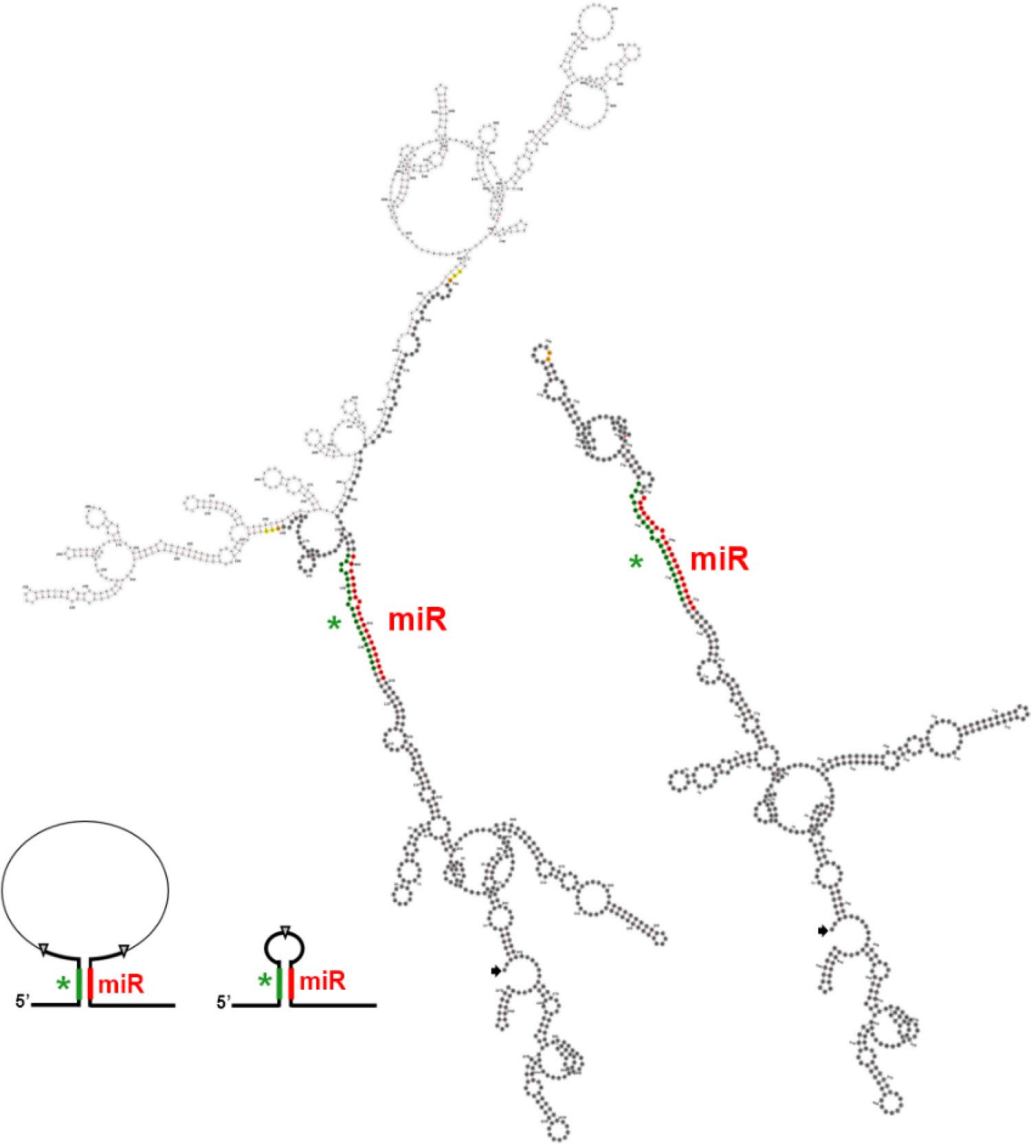
pri-miRNAtop14 secondary structure and schematic representation of the resulting miRNA hairpin, spliced (right), and non-spliced (left) variants (*Solanum lycopersicum).* pri-miRNAtop14 secondary structure was predicted by RNAfold (Lorenz et al. 2011) as the one with minimum free energy from the putative pri-miRNA sequence and visualizations were created using *forna* tool (Kerpedjiev et al. 2015). miRNAtop14 and miRNAtop14* are indicated in red and green, respectively, in both the schemes and the secondary structure representation. In the schemes, exons are represented as bold lines, introns as thin lines, and SS and exon–exon junctions as grey triangles. In the secondary structure representations, nucleotides belonging to an exon are dark grey, while the ones within the intron are light grey. GU-AC dinucleotides at the SS are coloured yellow. First exon 3′ end nucleotide and last exon 5′ end nucleotide are coloured orange to mark the exon–exon junction after splicing. The first 5′ residue of each transcript is indicated with a black arrow and the structures are always oriented with the miRNA stem-loop at the top

To confirm the expression and length of the primary miRNA transcript, we carried out an oligo(dT)-primed reverse-transcription followed by PCR and cloned and sequenced the PCR products. We found that there were in fact two polyadenylated transcripts: one full-length transcript and another one lacking most of the genomic sequence in between the miRNA-5p and miRNA-3p. After a BLAST search of NCBI Transcript Reference Sequences (Sayers et al. 2022) of *S. lycopersicum,* we found that there was a predicted transcript supported by mRNA and EST evidence that included both miRNA-5p and miRNA-3p but which was lacking the same stretch of nucleotides as our shorter PCR product (*S. lycopersicum* uncharacterized LOC101267134, NCBI reference sequence XR_182935.5). We hypothesised that the region missing from the shorter product was an intron, and indeed, we found the canonical GU-AC motif at the expected splice site. We concluded that the unusually long initial pri-miRNA transcript contained an intron that is spliced, allowing the formation of a canonical pri-miRNA hairpin that could be processed by Dicer. The predicted secondary structures for the pri-miRNAs before and after splicing supported this hypothesis (Fig. 2).

### miRNA and its intron–exon structure are conserved across Solanales

**Fig. 3:**
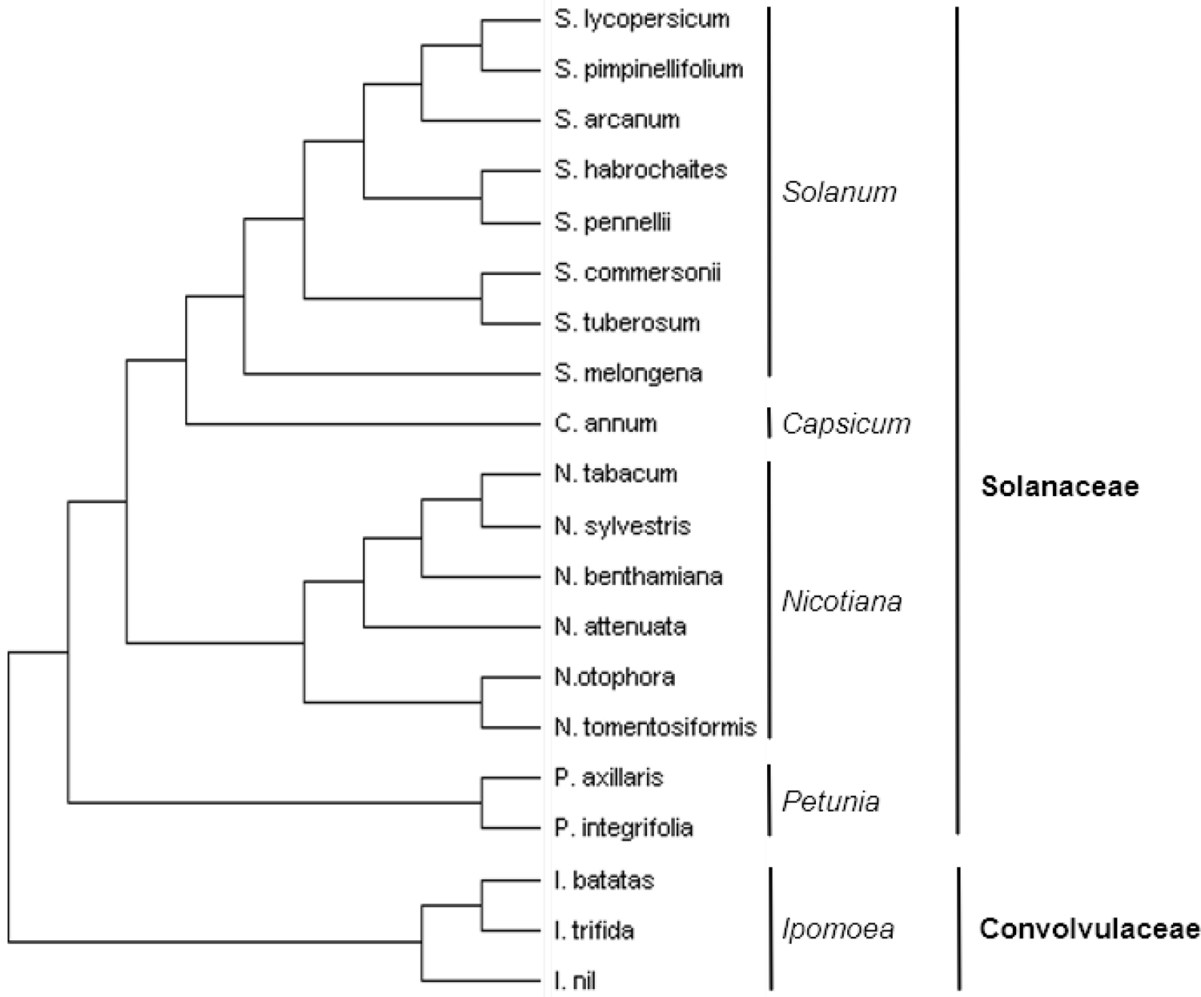
Cladogram depicting the evolutionary relationship among all species in which *MIRtop14* has been identified in NCBI database (Sayers et al. 2022). All of them belong to the Solanales order. Data extracted from several studies of the phylogeny of these species (Aoki and Ito 2000; Doganlar et al. 2002; Clarkson et al. 2004; Olmstead et al. 2008; Eserman et al. 2014; Aversano et al. 2015; Bombarely et al. 2016; Pease et al. 2016)

We investigated if this miRNA was present in other plants beyond *S. lycopersicum*. We carried out BLAST searches in various publicly available databases (Fernandez-Pozo et al. 2015; Hirakawa et al. 2015; Sayers et al. 2022) and found pre-miRNAtop14 in all of the four most studied Solanaceae genera: *Solanum*, *Capsicum*, *Nicotiana*, and *Petunia*. Beyond Solanaceae, we found this *MIR* to be present in its closest family, Convolvulaceae (Fig. 3 and Suppl. Table S3). The lack of genomic sequences made it impossible to determine if the gene was also present in any other families within the Solanales order. We finished our search for the *MIR* gene looking into the three orders closer to Solanales: Gentianales, Lamiales, and Boraginales. We could not find the mature miRNA sequence in any of them despite that one Gentianales species (*Coffea cenophora*) and one Lamiales species (*Mimulus guttatus*) have both high-quality draft genomes available. These results would indicate that this *MIR* arose at some point at the beginning of Solanales evolution, making it a relatively young *MIR*. Consistent with this, genomic data indicated that it appears in a single copy per genome in all species examined, unlike highly conserved miRNAs that tend to form multicopy gene families (Axtell and Bowman 2008).

**Fig. 4:**
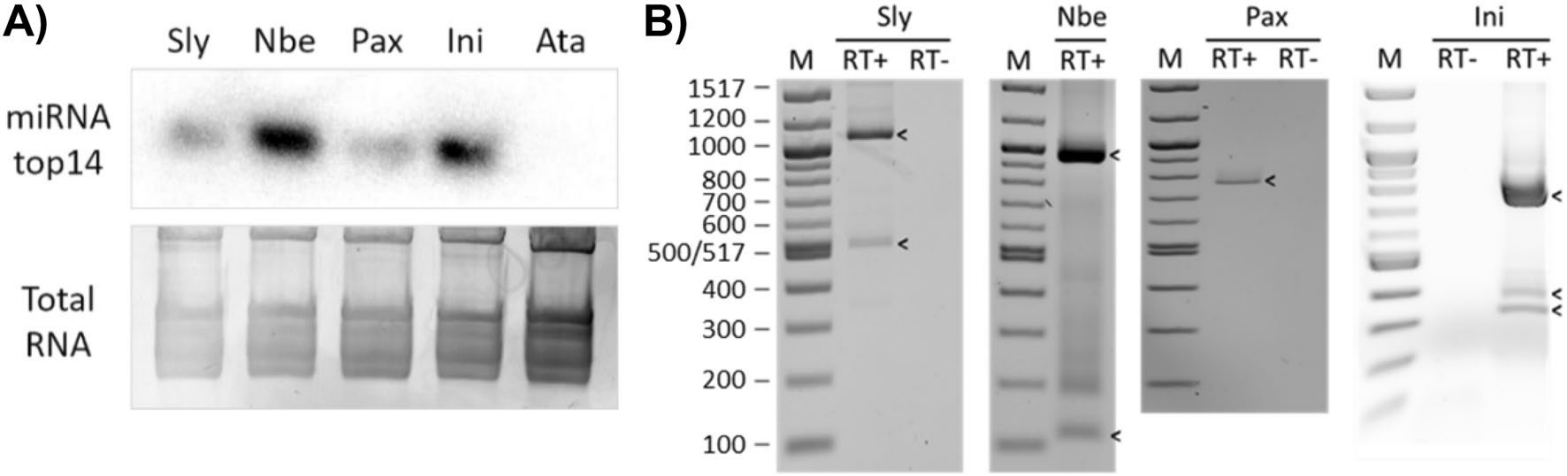
Detection of miRNAtop14 and pri-miRNAtop14 in different plant species. **A** Detection of mature miRNAtop14 in *Arabidopsis thaliana* (Ata) and four species of Solanales: *Solanum lycopersicum* (Sly), *Nicotiana benthamiana* (Nbe), *Petunia axillaris* (Pax), and *Ipomoea nil* (Ini) by northern blot. Ethidium bromide stained total RNA is included as loading control. **B** Detection of pri-miRNAtop14 after total RNA reverse transcription in Sly and Pax, and mRNA reverse transcription in Nbe (RT+) by PCR. Controls without reverse transcriptase enzyme were included to rule out genomic DNA contamination where indicated (RT−). Bands showing the expected length are marked by an arrowhead. Expected amplicons length from each species in nucleotides: Sly = 1071 and 503, Nbe = 941 and 111, Pax = 760, Ini = 788, 359, and 295. M, 100 bp marker; the size of the bands is given in base pairs (bp)

We then extended our search to assess the conservation of the intron and the alternative transcripts. We found that there was a distance of more than 600 bp between miRNA-5p and miRNA-3p in *Solanum*, *Capsicum*, and *Nicotiana* species and transcripts predicted to harbour an intron in between the hairpin arms in some of them (Suppl.Table S4). This suggests that the intron found in *S. lycopersicum* is conserved in these three genera. On the contrary, in *Petunia*, miRNA-5p and miRNA-3p were a few nucleotides apart creating a canonical hairpin without any intron. Surprisingly, in *Ipomoea*, the genera that has diverged earlier from the rest, the distance that separated miRNA-5p and miRNA-3p was around 450 bp, again longer than expected. We decided to experimentally test this information in four of the Solanales genera, using the species *S. lycopersicum, Nicotiana benthamiana, Petunia axillaris*, and *Ipomoea nil*. First, we confirmed that mature miRNA was produced in all four species by a small RNA northern-blot analysis and also verified that no mature miRNA was present in the distant species *Arabidopsis thaliana*, that was used as negative control (Fig. 4A).

We subsequently performed oligo(dT) reverse-transcription, PCR, and cloning to determine the pri-miRNAs that were being transcribed in each species. As expected, in *N. benthamiana*, two transcripts similar to the ones seen in *S. lycopersicum* were detected, while in *P. axillaris*, a single transcript was amplified. In *I. nil*, besides two transcript variants similar to the ones identified in *S. lycopersicum* and *N. benthamiana*, a third transcript was detected. This transcript had the same 5’ splicing site as the usual spliced variant, but its 3’ splicing site was further downstream, so the miRNA sequence was spliced out. This third transcript is supported by NCBI sequencing data, while the other spliced transcript that forms the miRNA hairpin had not been reported (Sayers et al. 2022) (Fig. 4B). These results confirm the production of this miRNA in Solanaceae and Convolvulaceae family members and the conservation of an alternatively spliced pri-miRNA in several of its genera.

### Accumulation of mature miRNA in *S. lycopersicum* is influenced by its intron

After confirming that this *MIR* was in fact producing a spliced and a non-spliced transcript in both *S. lycopersicum* and other relatives, we investigated whether both transcripts were being processed into miRNA despite the unusually long stem loop in one of them. To further explore the possible influence of the intron–exon structure in the biogenesis of the miRNA, we created three constructs expressing different pri-miRNA intron–exon structures, transformed them into *Arabidopsis*, and measured the accumulation of mature miRNA in each. One of the constructs contained the wild-type *MIR* gene and could produce both spliced and non-spiced transcript. A second construct harboured a version of the gene without the intron, producing only the intronless variant of the pri-miRNA. Finally, the third construct carried a full *MIR* gene including its intron sequence, but had a mutation at the 5′ splicing site that prevented splicing and limited the production of pri-miRNA to the non-spliced transcript form.

**Fig. 5:**
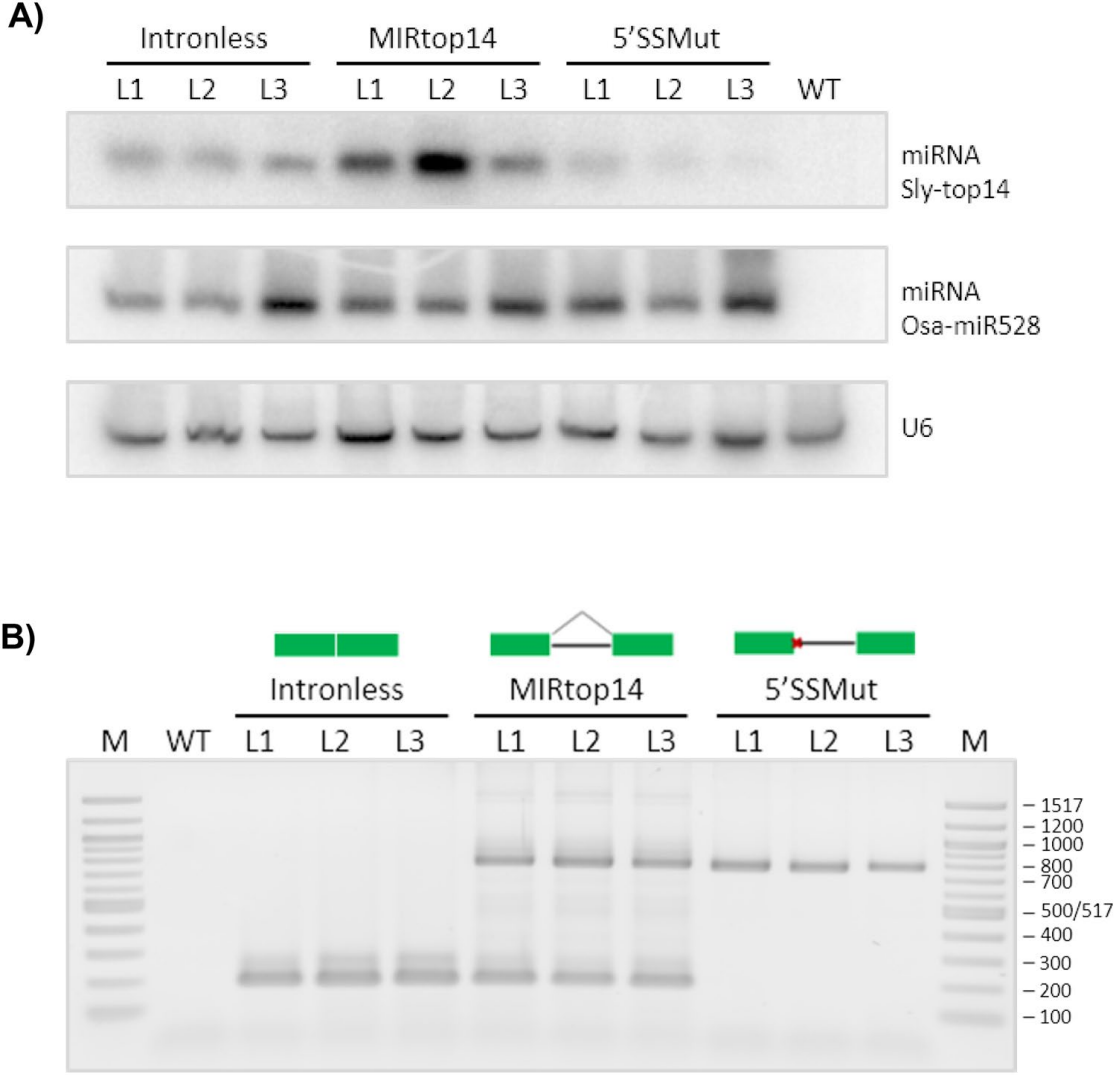
Effect of the intron in mature miRNAtop14 accumulation.** A** Northern-blot detection of mature miRNAtop14 in *A. thaliana* wild type (WT) and *A. thaliana* transformed with the three *MIRtop14* constructs (Intronless, *MIRtop14* and 5′SS MUT). Three independent transgenic lines (L1, L2 and L3) were analysed per construct. Osa-miRNA528, that was part of the T-DNA, detection was included as internal control of gene expression. U6 detection was included as loading control. **B** Detection of pri-miRNAtop14 in each sample after total RNA reverse transcription. Bands of 752 nucleotides correspond to the amplification of pri-miRNAtop14 with intron and bands of 184 nucleotides correspond to the amplification of pri-miRNAtop14 without intron. M, 100 bp marker; the size of the bands is given in base pairs (bp)

Levels of mature miRNA generated from each miRNA variant were measured by northern blot (Fig. 5). The construct with the non-spliceable gene produced an almost undetectable amount of mature miRNA compared with the other two constructs. This confirms that the unusual length of the pri-miRNA stem loop hinders the efficiency of miRNA dicing. The pri-miRNA has to first undergo splicing for miRNA processing to effectively take place. Although less marked, the accumulation of miRNA was also lower in the construct with the intronless gene than in the one carrying the wild type. This finding is in line with observations that introns adjacent to miRNA hairpins improve mature miRNA accumulation (Bielewicz et al. 2013; Schwab et al. 2013). Our experiment shows that introns in the middle of a miRNA stem-loop can also have this enhancement effect.

### miRNA cleaves LPR in Solanaceae family

**Fig. 6:**
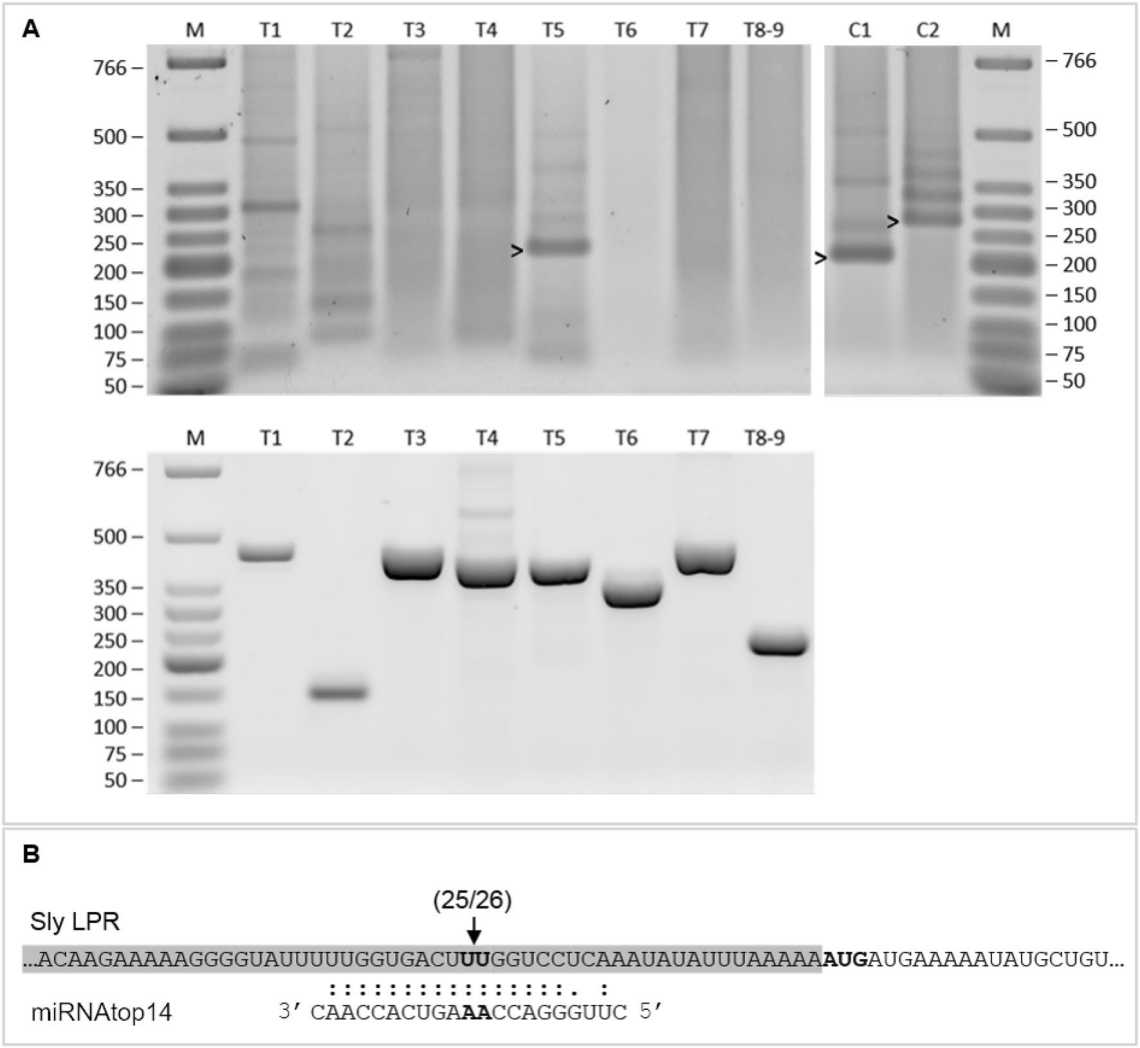
RLM-RACE analysis and miRNAtop14-*LPR* targeting in *Solanum lycopersicum.* **A** RLM-RACE analysis of the nine predicted targets predicted in *Solanum lycopersicum*, compiled in Table 1, each labelled with a target number (e.g., T1) according to Table 1. Target T8 and target T9 share the same sequence in the region of the putative miRNAtop14 cleavage, so they could not be independently analysed (they have the same primers), and therefore, their common product is labelled as T8–9. Top Nested PCR products from RLM-RACE run in an agarose gel. Two control targets are included: C1, LA cleaved by miRNA319 and C2, GRAS24 cleaved by miRNA171. Bands showing the expected length are marked by an arrowhead (T5, C1 and C2). Expected product length from each target in nucleotides: T1 = 77, T2 = 145, T3 = 114, T4 = 145, T5 = 197, T6 = 158, T7 = 203, T8–9 = 140, C1 = 209, and C2 = 274. T1 and T2 show bands that are proximate to the expected amplicon size, so these bands were recovered and cloned as well. T1 cloning failed, while T2 band resulted to be the product of an unspecific amplification. Bottom control of target mRNA presence in the sample; PCR amplification across putative miRNAtop14 directed cleavage. All targets are present in the sample in non-cleaved form. Expected product length from each target in nucleotides: T1 = 437, T2 = 149, T3 = 434, T4 = 405, T5 = 378, T6 = 348, T7 = 449, and T8-9 = 235. M, low-molecular-weight marker; the size of the bands is given in base pairs (bp). **B** miRNA-T5 (*LPR*) target site interaction scheme and results of the cloning and sequencing of the RLM-RACE products. Shadowed in grey 5′UTR followed by the translation start codon in bold. 25 out of 26 clones showed the cleaved position indicated by the arrow, which correspond to the expected cleaved position between miRNA nucleotides 10th and 11th (in bold)

Next, we predicted targets for the mature miRNAtop14. Using the psRNAtarget server (Dai and Zhao 2011), we obtained a list of 9 putative targets in the *S. lycopersicum* transcript cDNA library version 2.4, SGN (Fernandez-Pozo et al. 2015) (Table 1). All of them were subsequently analysed through RLM-RACE to determine if any was indeed being cleaved by the miRNA. From all nine transcripts, only the one coding for the LPR protein was confirmed to be specifically cut at the miRNA annealing site (Fig. 6).

**Table 1:**
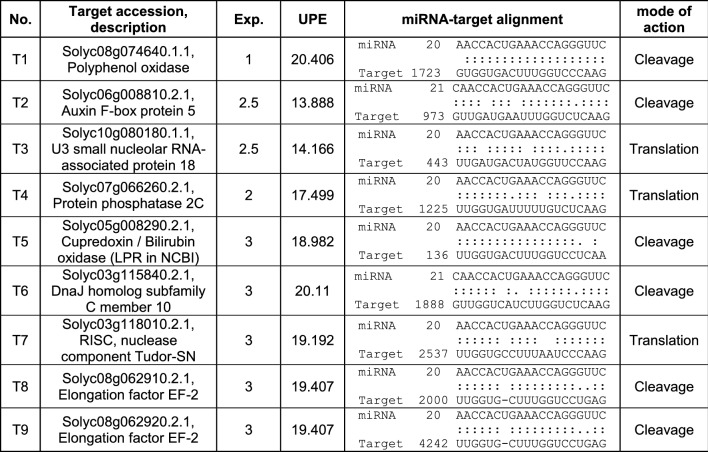
*Solanum lycopersicum* miRNAtop14 predicted targets by psRNAtarget server

Column 1 target number, given to identify each target in our RLM-RACE experiments. Column 2 target accession and description, according to SGN transcript cDNA library version 2.4. Column 3 expectation (Exp.), a score for miRNA-target complementarity, was set to a maximum of 3. Column 4 UPE, target accessibility as the maximum energy to unpair the target site, was set to a maximum of 25. Column 5 miRNA-target alignment gives the position of the first aligning nucleotide of the target and the last aligning nucleotide of the miRNA, considering the first position the 5′ end in both cases. The sequence of the target is written from the 5′ to the 3′ end (from left to right) and the sequence of the miRNA in the opposite direction. Column 6 miRNA mode of action was predicted to be cleavage whenever there were not mismatches between target and miRNA nucleotides 9 and 11, and translational repression otherwise

**Fig. 7:**
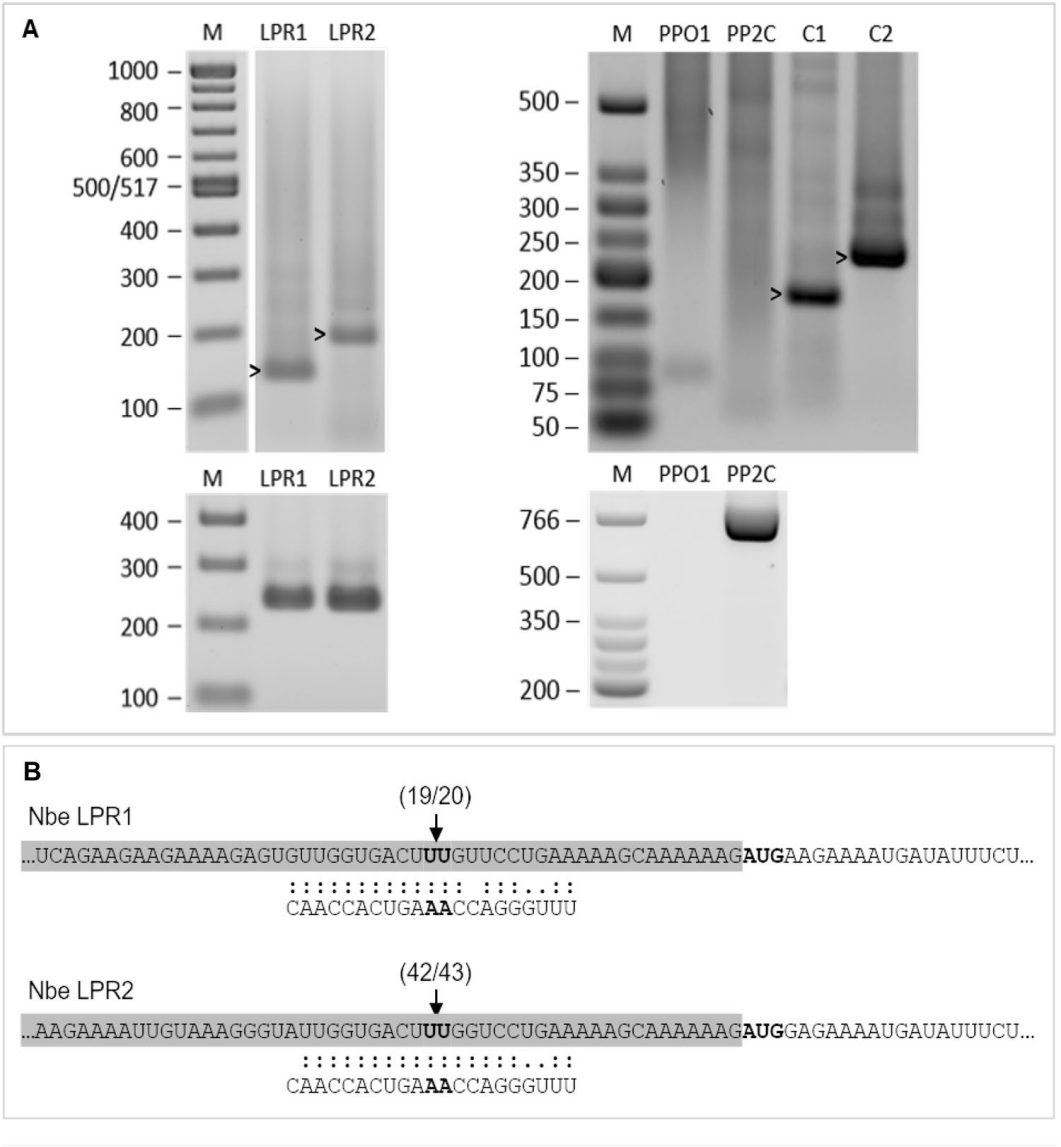
RLM-RACE analysis and miRNAtop14-*LPR* targeting in *Nicotiana benthamiana.* **A** RLM-RACE analysis of cleavage by miRNAtop14 of its *LPR1*, *LPR2*, *PPO1*, and *PP2C* predicted targets in *N. benthamiana*, shadowed in grey in Suppl. Table S5. Top nested PCR products from RLM-RACE run in an agarose gel. Two control targets are included: C1, TCP4 cleaved by miRNA319 and C2, SCL6 cleaved by miRNA171. Bands showing the expected length are marked by an arrowhead (LPR1, LPR2, C1, and C2). Expected product length from each target in nucleotides: LPR1 = 125, LPR2 = 179, PPO1 = 145, PP2C = 236, C1 = 182, and C2 = 237. Bottom control of target mRNA presence in the sample; PCR amplification across putative miRNAtop14 directed cleavage. All targets but PPO1 are present in the sample in non-cleaved form. Expected product length from each target in nucleotides: LPR1 = 232, LPR2 = 233, PPO1 = 563, and PP2C = 778. M, 100 bp marker for LPR1 and LPR2 gels, low-molecular-weight marker for PPO1 and PP2C gels; the size of the bands is given in base pairs (bp). **B** miRNA-*LPR1* and *LPR2* target site interaction schemes and results of the cloning and sequencing of the RLM-RACE products. Shadowed in grey 5′UTR followed by the translation start codon in bold for both transcripts. 19 out of 20 clones in *LPR1* and 42 out of 43 clones in *LPR2* showed the cleaved position indicated by the arrows, which correspond to the expected cleaved position between miRNA nucleotides 10th and 11th (in bold)

We carried out the same in silico analysis using the *N. benthamiana* transcript library Niben101, SGN (Fernandez-Pozo et al. 2015) and a total of 22 putative targets were obtained (Suppl. Table S5). Among them, the two with higher expectation corresponded to the two *N. benthamiana* LPR transcripts. Both variants were confirmed to be cleaved by the miRNA through RLM-RACE (Fig. 7). Besides LPR, polyphenol oxidase (PPO), and protein phosphatase 2C (PP2C), several protein members were identified as possible targets. Both protein families were identified as putative targets in *S. lycopersicum*, although RLM-RACE analysis did not detect their cleavage. One transcript of each family was also analysed by RLM-RACE in *N. benthamiana*, but again no correct cleavage product was found.

After these results, we decided to test whether LPR was also targeted by miRNAtop14 in *Petunia* and *Ipomoea* using once again psRNAtarget (Dai and Zhao 2011). We compared miRNA against the LPR one or two variants found in *P. axillaris, P. integrifolia, I. trifida,* and *I. nil*. Targeting was not predicted in any of them (Suppl.Table S6). That came as a surprise, since the miRNAtop14 sequence was very conserved in these two genera. We carried out an alignment between LPR and miRNA in which no complementarity was found in *Ipomoea* and only partial complementarity was found in *Petunia*. Although not predicted, we tested any remote possibility of cleavage in *P. axillaris* by RLM-RACE, but it was not detected.

These results prove that the miRNA targets LPR in *Solanum* and *Nicotiana* genera. Interestingly, they also show that this target must have been newly acquired after the divergence of these genera despite this miRNA being conserved from before.

### miRNAtop14 level does not change during phosphate starvation

The expression level of LPR responds to phosphate deprivation (Svistoonoff et al. 2007); therefore, it was hypothesised that the expression level of miRNAtop14 would be negatively correlated with the level of its target. The accumulation level of miRNAtop14 during phosphate starvation was investigated by northern blot in three different tissues (root, stem, and leaf), but there was no significant difference in the level of mature miRNAtop14 between plants grown in the presence or absence of phosphate and during a replenishment time course (Suppl. Fig. S2). The control, miR399 that is known to respond to phosphate level, showed the expected increase in plants grown in the absence of phosphate compared to plants grown in full media and also showed the expected decrease after replenishment the media with phosphate.

Table 1*Solanum lycopersicum* miRNAtop14 predicted targets by psRNAtarget server. Column 1 Target number, given to identify each target in our RLM-RACE experiments. Column 2 Target accession and description, according to SGN transcript cDNA library version 2.4. Column 3 Expectation (Exp.), a score for miRNA-target complementarity, was set to a maximum of 3. Column 4 UPE, target accessibility as the maximum energy to unpair the target site, was set to a maximum of 25. Column 5 miRNA-target alignment gives the position of the first aligning nucleotide of the target and the last aligning nucleotide of the miRNA, considering the first position the 5’ end in both cases. The sequence of the target is written from the 5’ to the 3’ end (from left to right) and the sequence of the miRNA in the opposite direction. Column 6 miRNA mode of action was predicted to be cleavage whenever there were not mismatches between target and miRNA nucleotides 9 and 11, and translational repression otherwise.

## Discussion

We identified a new miRNA in *S. lycopersicum* that could not be identified through the standard workflow. The first step in analysing sRNA sequencing data is to map the reads to the genome, and then, miRNA prediction programs generate RNA secondary structures for the flanking region. Usually, two structures are predicted, one with a longer upstream flanking region and another with a longer downstream region, as the read could be either the miRNA-5p or the miRNA-3p (Paicu et al. 2017). These programs usually fold 300–400 nts, because the vast majority of pri-miRNAs are of that length. However, the pri-miRtop14 contains an intron; therefore, its length is about 700 nts and therefore, it would not be predicted as a miRNA, because only 300–400 nts around the mature miRNAtop14 do not fold into a proper hairpin structure. The reason we were able to identify it was that there was no genome sequence available at the time; therefore, we could not map the reads. All reads were searched against each other to find potential reverse complementary miRNA-5p and miRNA-3p sequences. This approach identified miRtop14-5p and miRtop14-3p as potential miRNA sequences and northern blot analysis confirmed the accumulation of miRtop14-3p as a 21 nt RNA species. RT-PCR experiments confirmed the accumulation of a shorter pri-miRNA sequence with the expected hairpin structure and the presence of the canonical splicing motif supported the idea that an intron is removed from the initial pri-miRNA. Baksa et al. (2015) also identified the same miRNA in a high-throughput study of *N. benthamiana* sRNAs for similar reasons. The *N. benthamiana* genome has just been sequenced (Ko et al. 2024); therefore, Baksa et al. could only use an EST database to map the reads, and therefore, they were able to map the miRNA-5p and miRNA-3p to the spliced pri-miRNA. However, due to the lack of a genome sequence and further analysis, it was not recognised that the *N. benthamiana* pri-miRNA contains an intron.

Since it is unusual that an intron is situated between miRNA-5p and miRNA-3p, we investigated how conserved miRtop14 was. The mature miRNA sequence (miRtop14-3p) was very conserved among Solanales species within *Solanum*, *Capsicum*, *Nicotiana*, *Petunia,* and *Ipomoea* genera. Besides, pri-miRNA secondary structure analysis predicted the pairing of miRNA-5p and miRNA-3p sequences in all secondary structures of all species studied. Furthermore, northern-blot analyses showed that all species tested ubiquitously produced mature miRNA at highly detectable levels. All these results suggest that this miRNA must have a biological function which is preserving it through evolution. The unusual feature of having an intron between miRNA-5p and miRNA-3p have been confirmed in *Solanum, Nicotiana,* and *Ipomoea* genera, while the intron was missing in *Petunia*. This finding suggests that the ancestral *MIR* already had a long stretch of DNA between miRNA-5p and miRNA-3p corresponding to the current intron that was lost in *Petunia* once rather than gained twice independently in both the *Ipomoea* and *Solanum–Capsicum–Nicotiana* branches.

Although it is not known whether mature miRNA expression is regulated through splicing, it has been proved that the presence of the intron itself is indeed influencing mature miRNA accumulation. Besides the need for the intron to be spliced for effective miRNA production, we were able to confirm that the pri-miRNA with the intron had enhanced miRNA biogenesis relative to an intronless version of the transcript. This surprising finding had already been observed with introns downstream to miRNA hairpins which increased mature miRNA accumulation (Bielewicz et al. 2013; Schwab et al. 2013). Since introns upstream did not show this effect, it was then hypothesized that the factors mediating the increase in miRNA accumulation should be acting either before or during spliceosome recruitment (Schwab et al. 2013). Our results are in line with those studies and shows for the first time that introns in the middle of a miRNA stem-loop can also have this enhancement effect. They also support the hypothesis that the mediating mechanism should be acting simultaneously to spliceosome recruitment, rather than before.

Introns are known to play different roles in regulating the expression of proteins from spliced mRNAs. Delayed splicing from intron-retaining transcripts serves to delay translation (Boothby et al. 2013). There are also examples of simultaneous production of functional and non-functional alternatively spliced mRNAs, which fine-tune the amount of protein produced (Filichkin et al. 2015). Any of these scenarios could be easily extrapolated to a miRNA system: instead of translation, dicing would be delayed; instead of protein amount, mature miRNA accumulation would be regulated.

In silico search for the target of miRtop14 yielded several possible transcripts in both *S. lycopersicum* and *N. benthamiana*. LPR cleavage prediction by this miRNA was confirmed experimentally using RLM-RACE in *S. lycopersicum* and among paralogous LPR variants in *N. benthamiana*. Additionally, we could detect LPR cleavage in published *S. lycopersicum* degradome data (Lopez-Gomollon et al. 2012). LPR has a known role in phosphate starvation in *Arabidopsis* and rice (Svistoonoff et al. 2007; Müller et al. 2015; Cao et al. 2016; Ai et al. 2020); however, its role in *S. lycopersicum* remains unknown. Although the role of LPR in *S. lycopersicum* has not been studied and established, it is reasonable to assume that it is similar to the role identified in *Arabidopsis* and rice. LPR plays a role to adaptation to low phosphate level in those species, and its expression level increases during phosphate deprivation (Svistoonoff et al. 2007; Müller et al. 2015; Cao et al. 2016; Ai et al. 2020). However, we did not find any evidence that the accumulation of the mature miRNAtop14 responds to low level of phosphate. This seems contradictory, because a general assumption is that the level of miRNAs negatively correlates with the expression level of their target mRNA. However, this is not always the case. It has been shown that the expression of miR395, that plays an important role in sulphur assimilation (Kawashima et al. 2011), shows a positive correlation to the expression level of its target SULTR2;1 as both miR395 and SULTR2;1 are upregulated during sulphur starvation (Kawashima et al. 2009). The explanation for this is that miR395 is predominantly expressed in phloem companion cells, while SULTR2;1 is mainly expressed in the xylem (Takahashi et al. 1997). We also found that the lack of negative correlation between the expression level of miRNAs and their targets is more widespread and not specific to miR395 and SULTR2;1 (Lopez-Gomollon et al. 2012). Further experiments are required to study the spatial expression pattern of miRNAtop14 and LPR and also the biological significance of the cleavage of LPR mRNA by miRNAtop14.

Interestingly, LPR targeting was neither predicted nor observed experimentally in genera further apart such as *Petunia* and *Ipomoea* despite the high conservation of the miRNA sequence and its hairpin in all these genera. This observation raises the possibility of miRNAtop14 having another target in *Petunia* and *Ipomoea*, a target which may precede LPR evolutionarily and that could be shared by all species. In this scenario, LPR would be a later, additionally gained target with a specific role in *Nicotiana, Solanum,* and possibly *Capsicum* species. However, it is also possible that miRNAtop14 is such a young miRNA that it does not yet have a target in *Petunia* and *Ipomoea,* as young miRNAs often do not have targets (Rajagopalan et al. 2006; Fahlgren et al. 2007). In this scenario, miRNAtop14 only acquired a target in *Nicotiana, Solanum,* and possibly *Capsicum* species. There were two other promising potential targets, mRNAs encoding for PPO and PP2C proteins, since both have members predicted to be targeted by the miRNA in *Ipomoea* and *Petunia* as well as in *Solanum, Capsicum, and Nicotiana*. Also, they showed perfect complementarity to miRNAtop14. Furthermore, *PPO1* transcript was identified by Baksa et al. (2015) as a possible target of miRNAtop14 in *N. benthamiana* based on their degradome data. However, we could not confirm the cleavage of PPO1 in *N. benthamiana* given that our samples did not contain the transcript. Nevertheless, PPO was not detected to be cleaved in PPO containing *S. lycopersicum* samples and neither was detected the cleavage of PP2C in any of the two species.

## Supplementary Information

Below is the link to the electronic supplementary material.

Supplementary file1 (DOCX 2338 KB)

## Data Availability

The paper does not include any large datasets but individual northern blots, etc. are available on request.

## Abbreviations

LPR: Low phosphate root
*MIRs*: MiRNA genes
