# Supplementary material for: An intron-split microRNA mediates cleavage of the mRNA encoded by low phosphate root in Solanaceae
Source: Planta. 2025 Jan 7;261(2):27. doi: 10.1007/s00425-024-04596-8 (PMC11706861; doi:10.1007/s00425-024-04596-8)
Supplement: Supplementary file 1 — Supplementary file1 (DOCX 2338 KB) [file 425_2024_4596_MOESM1_ESM.docx]

**An intron-split microRNA regulates LOW PHOSPHATE ROOT in Solanaceae**

Zahara Medina Calzada^1^, Runchun Jing^1^, Simon Moxon^1^, Hong Zhu^2^, Ping Xu^1,3^, Tamas Dalmay^1^

**1 School of Biological Sciences, University of East Anglia, Norwich Research Park, Norwich, UK**

**2 South China Botanical Garden, Chinese Academy of Sciences, Guangzhou 510650, China**

**3 Current address: Shanghai Engineering Research Center of Plant Germplasm Resource, College of Life Sciences, Shanghai Normal University, Shanghai 200234, China**


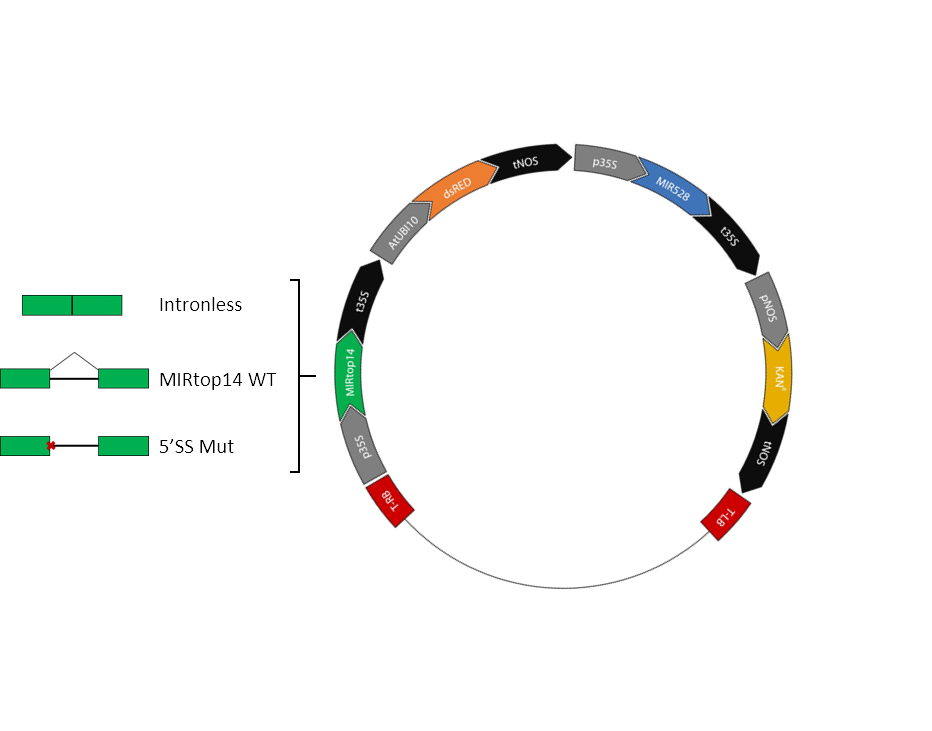


**A) B)**

**Suppl. Fig. S1** Scheme of the constructs used for *Arabidopsis* transformation. **A** Three different constructs only differing in *MIRtop14* sequence were created: one harbouring the wild type *MIRtop14* sequence with an spliceable intron (“*MIRtop14 WT*”, two green boxes separated by a black line), another one consisting in the two *MIRtop14* exons without intron (“Intronless”, two consecutive green boxes) and a final one similar to wild type *MIRtop14*, but with the 5’SS mutated from G/GT to C/CC making the intron non-spliceable (“5’SS Mut”, two green boxes separated by a black line and a red cross indicating the mutation at the 5’SS). **B** The rest of the plasmid apart from *MIRtop14* sequence was the same in all three constructs, consisting in four full transcriptional units in between the T-DNA right and left borders (“T-RB” and “T-LB” red squares in the image): *MIRtop14* sequence (green box) under CaMV 35S promoter and terminator (p35S and t35S grey boxes); dsRED sequence (orange box) under AtUBI10 promoter and NOS terminator (AtUBI10 and tNOS grey boxes); *MIR528* sequence (blue box) under CaMV 35S promoter and terminator (p35S and t35S grey boxes); KanR sequence (yellow box) under NOS promoter and terminator (pNOS and tNOS grey boxes).


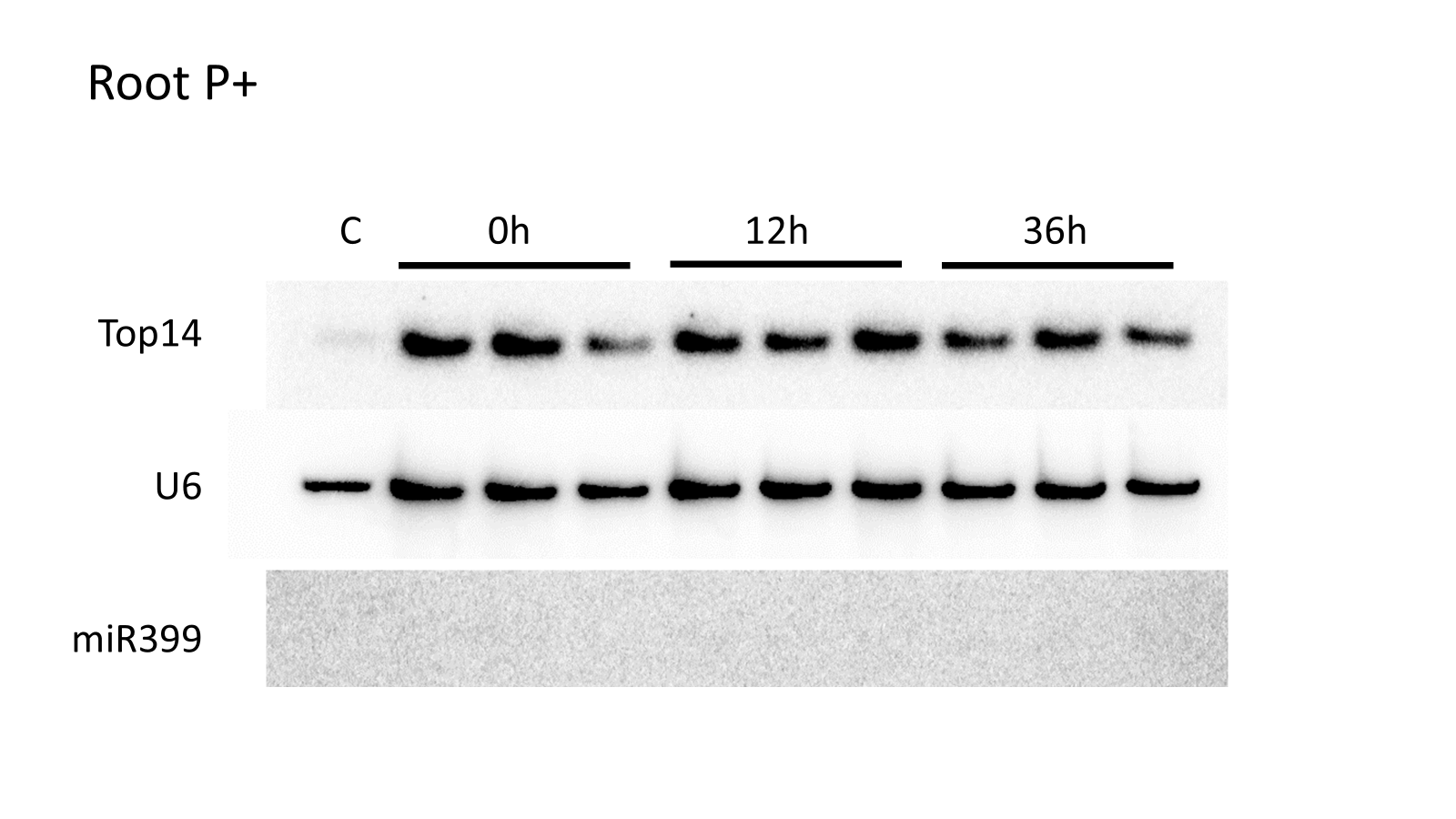

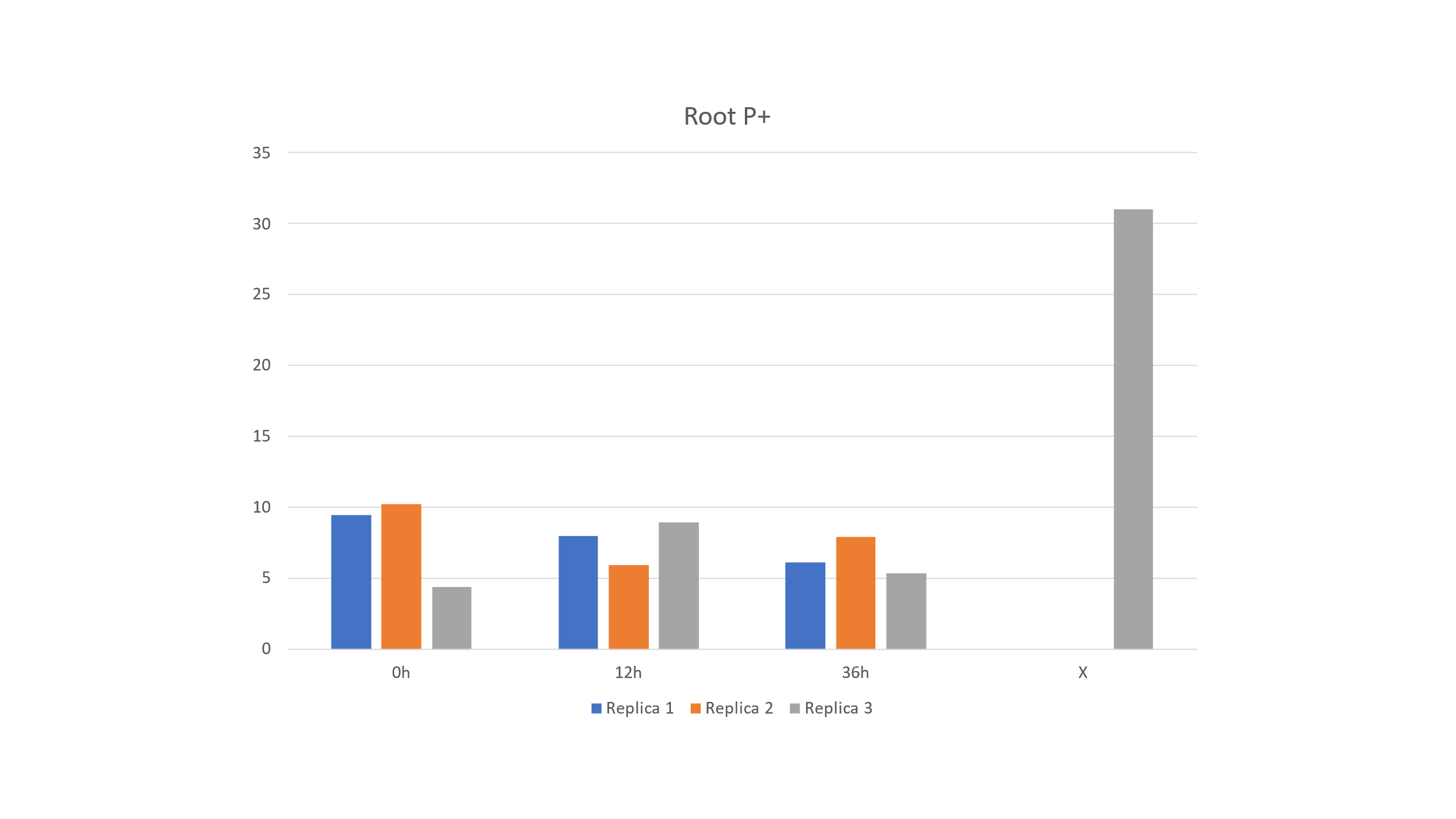


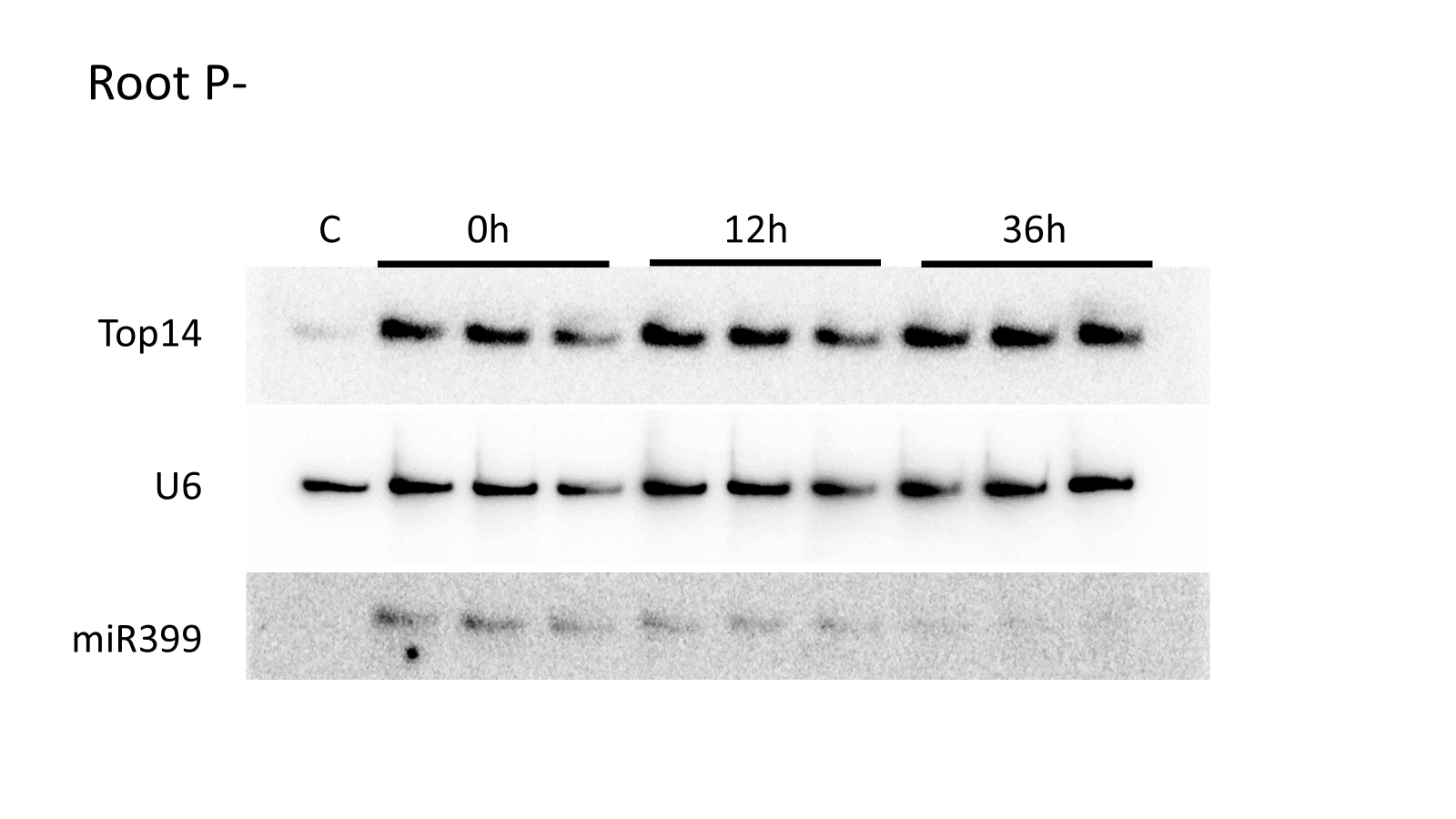

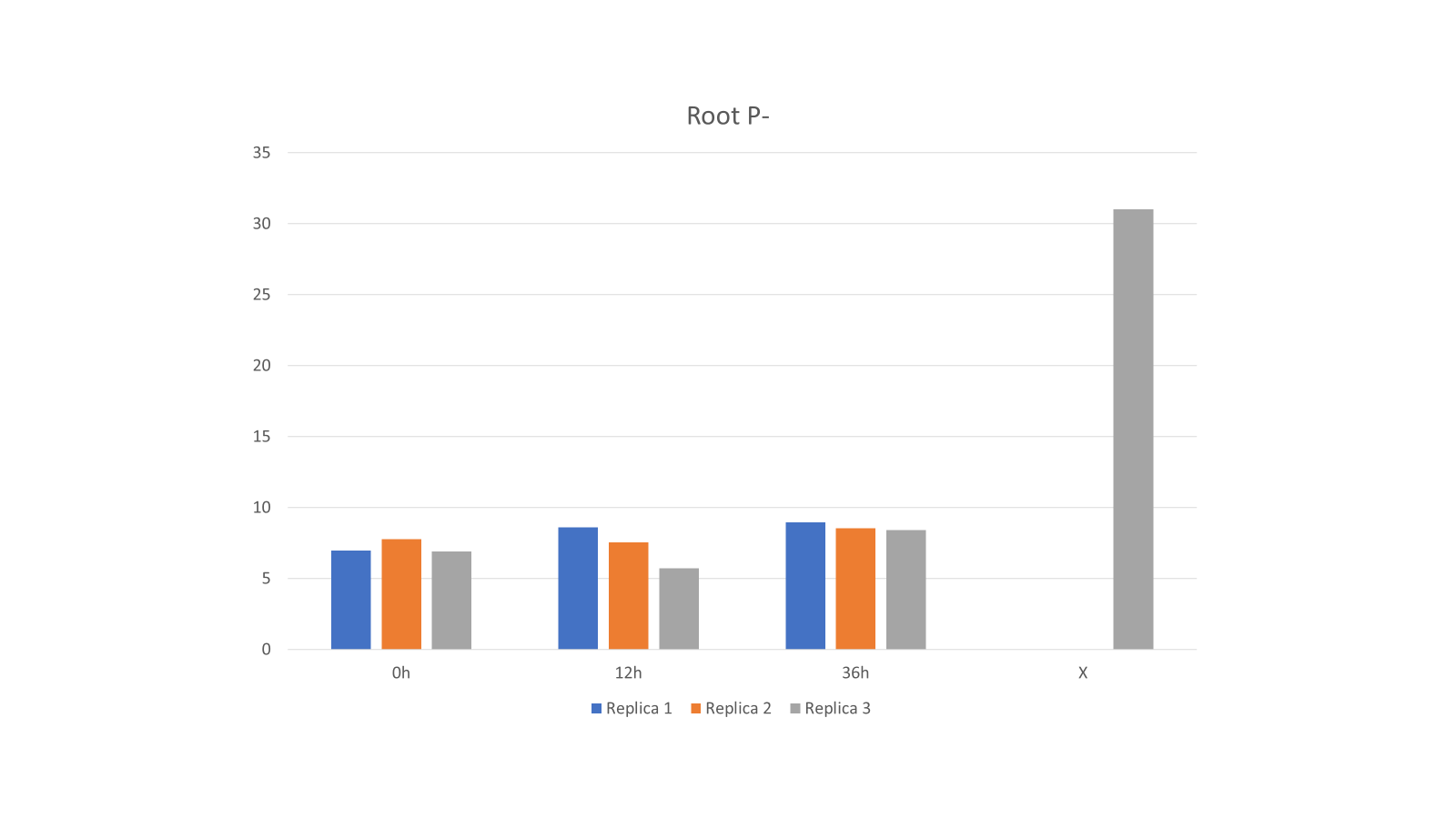


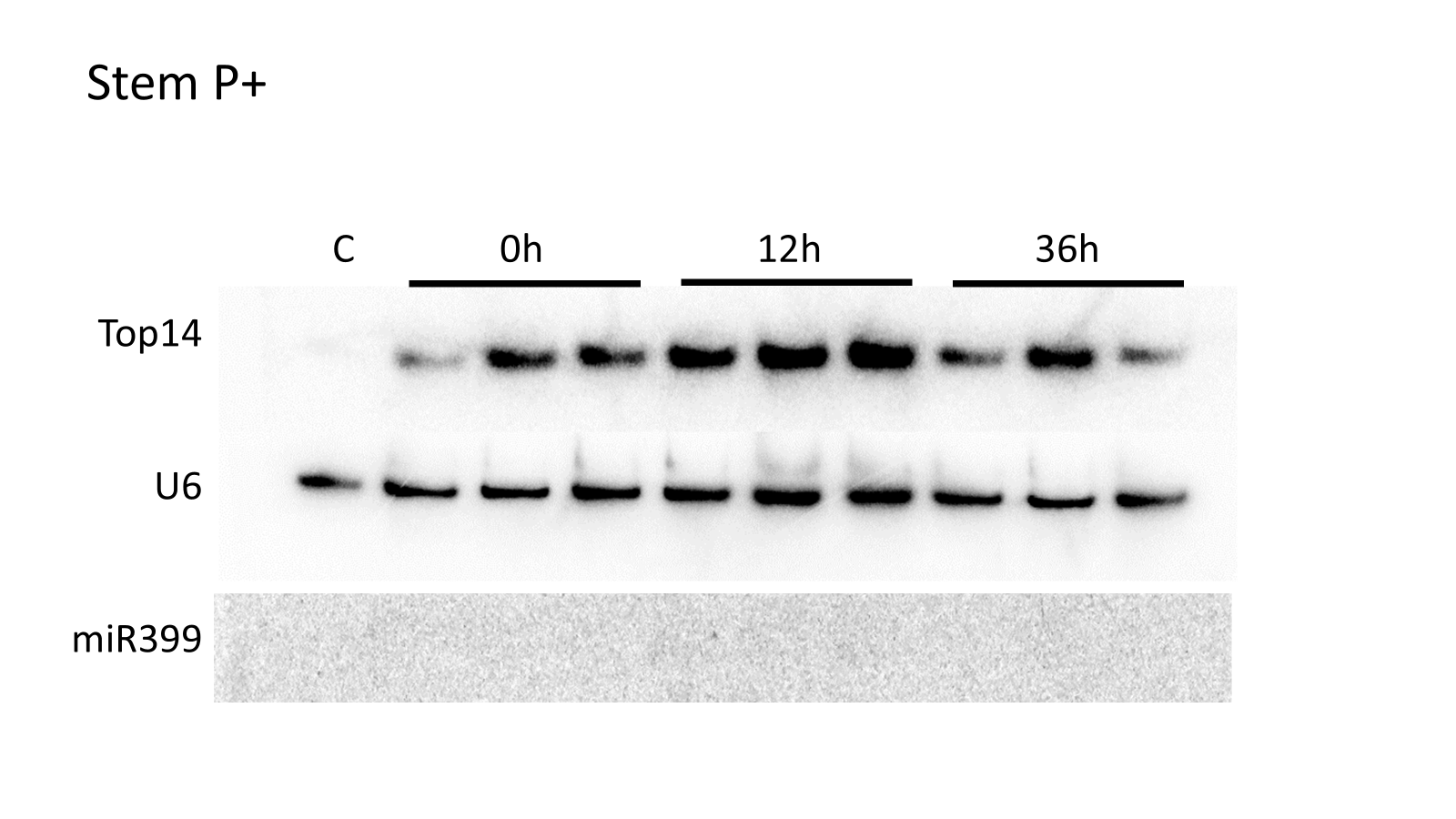

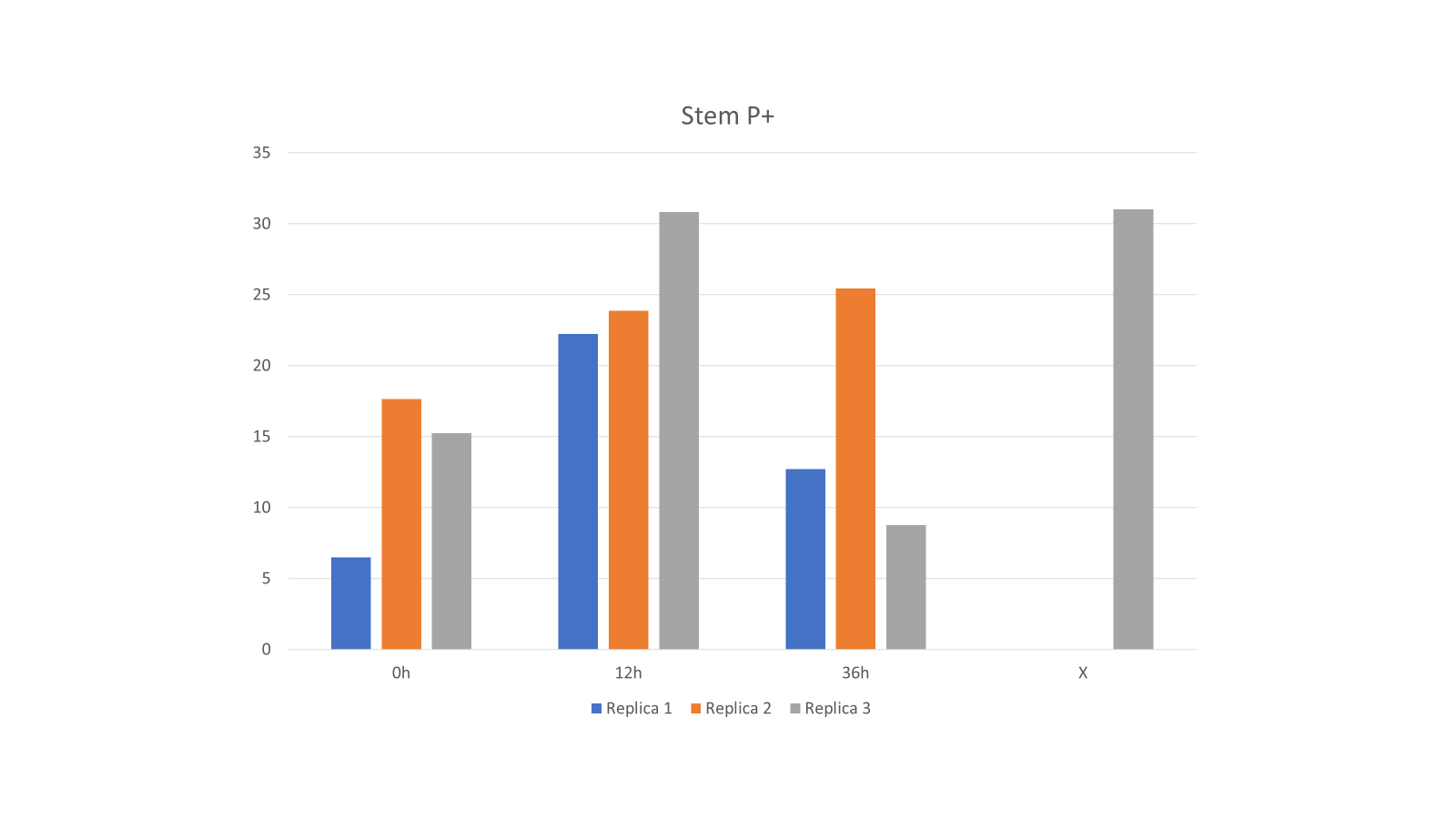


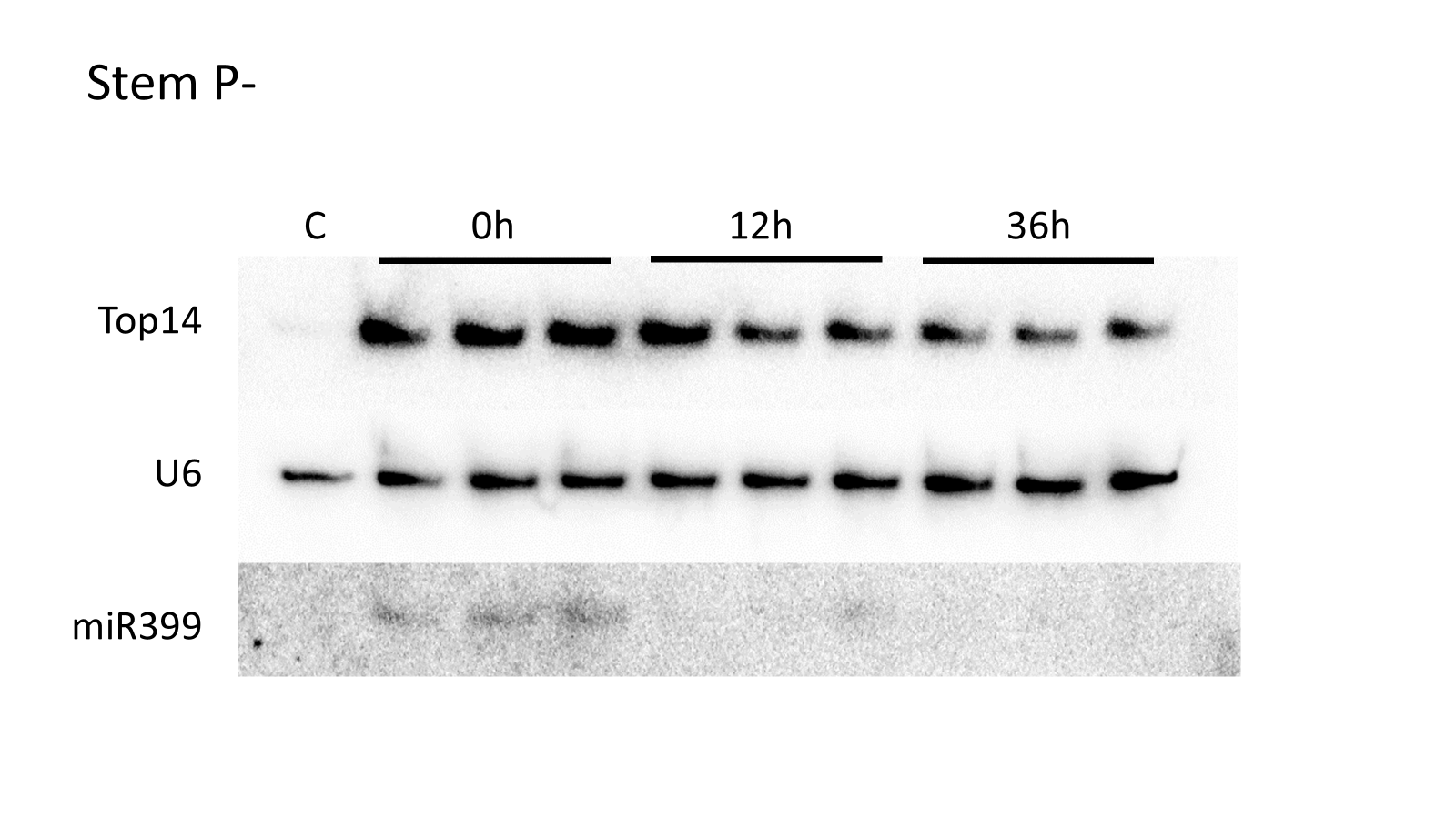

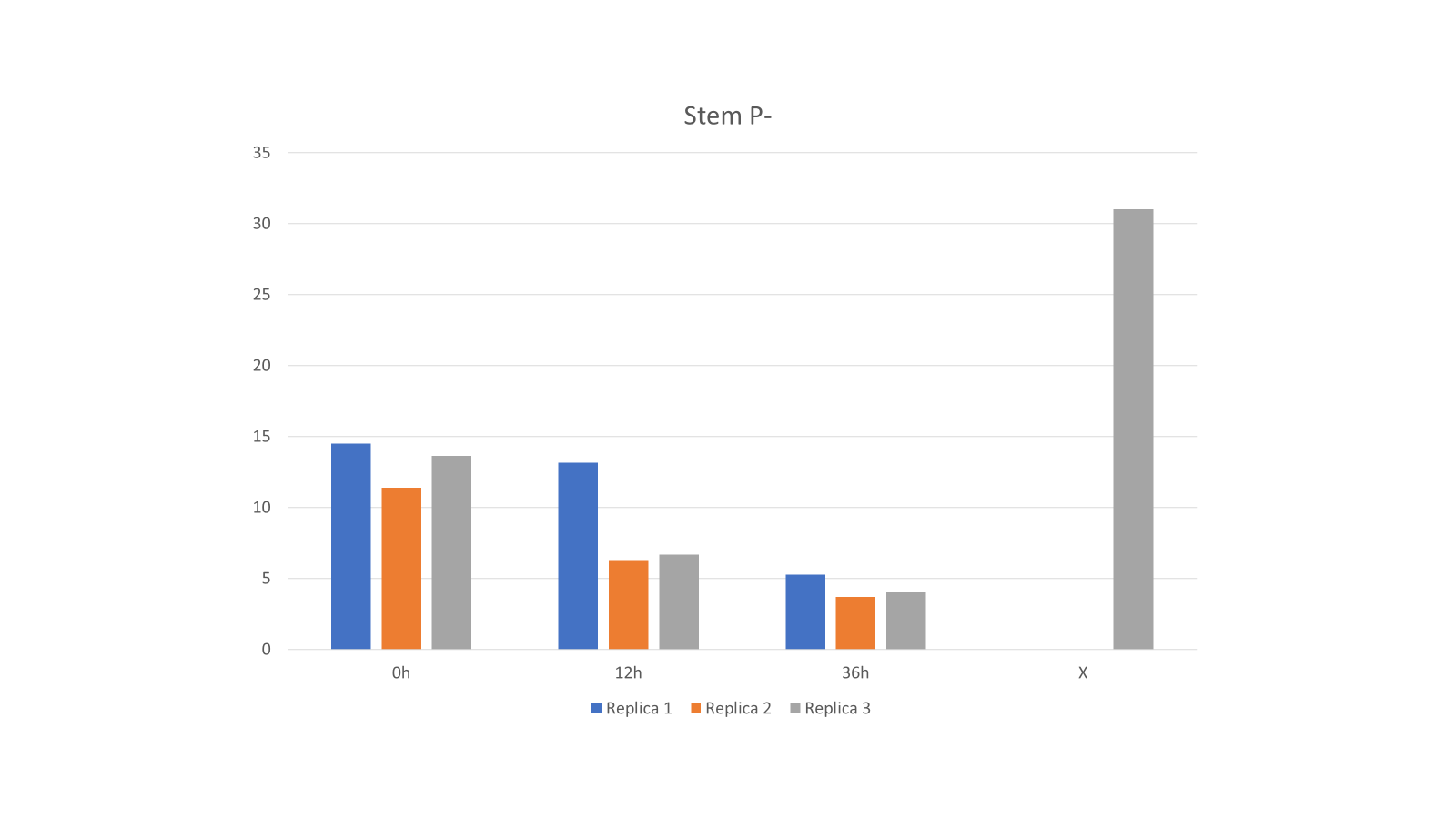


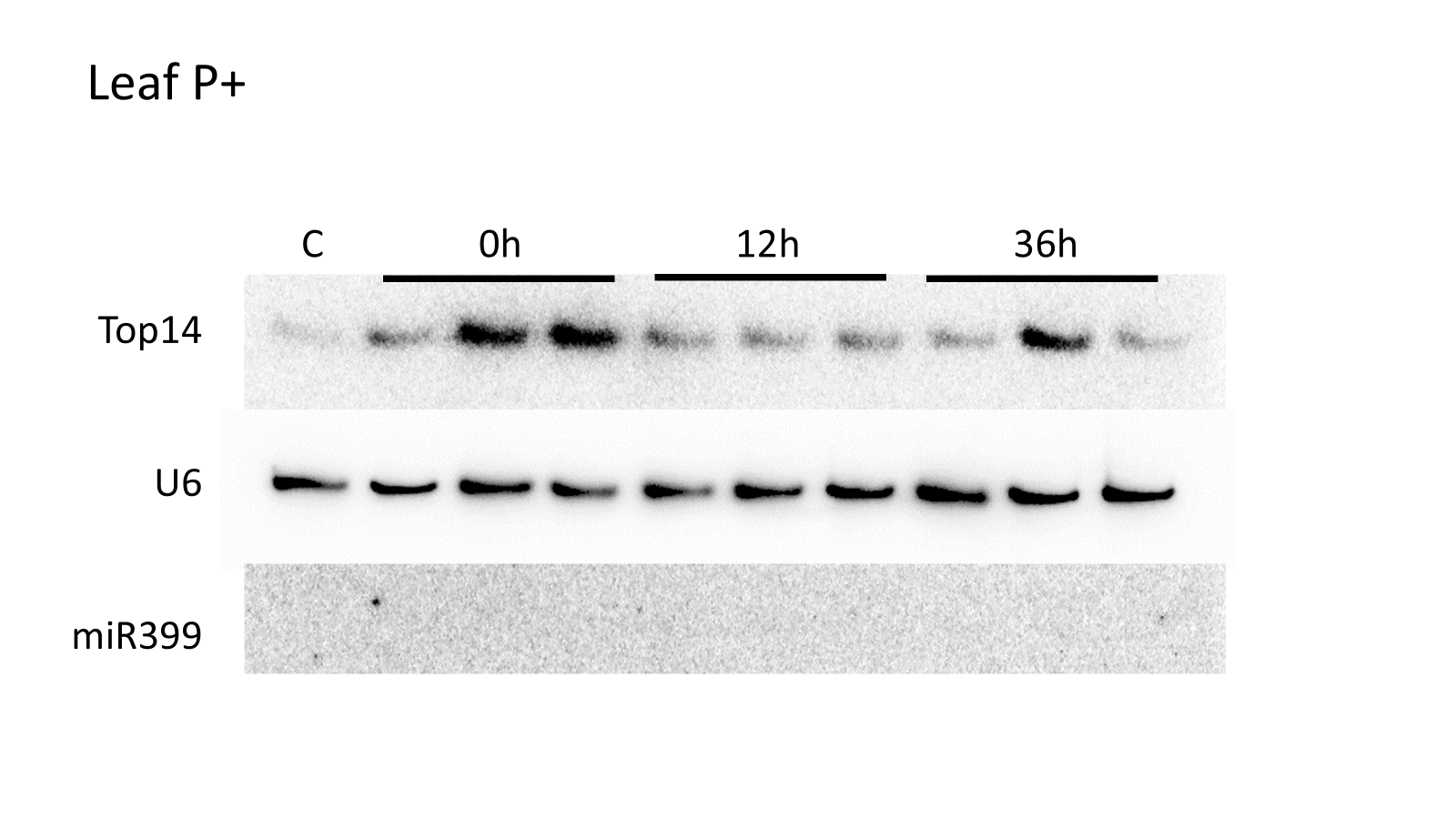

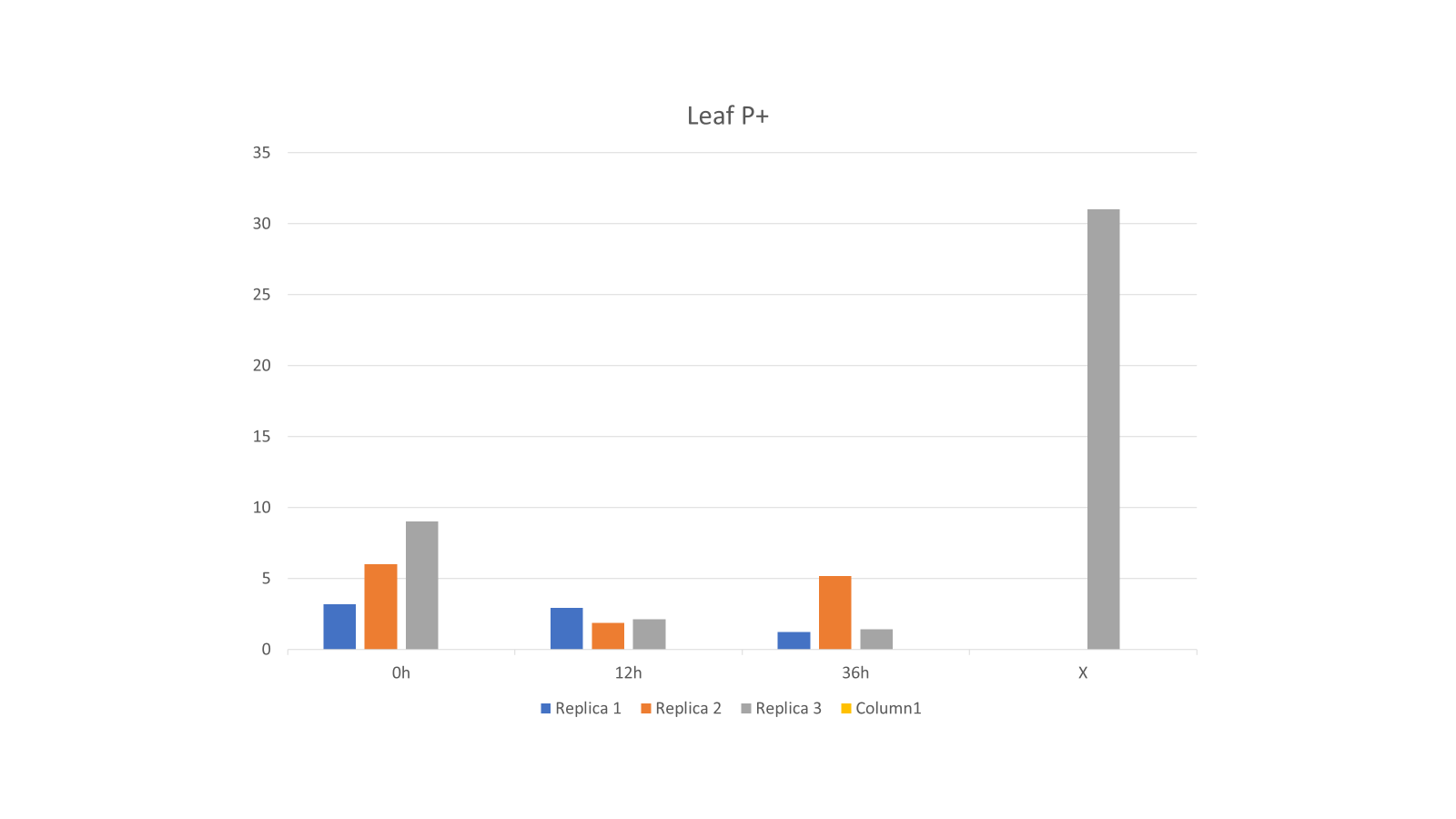


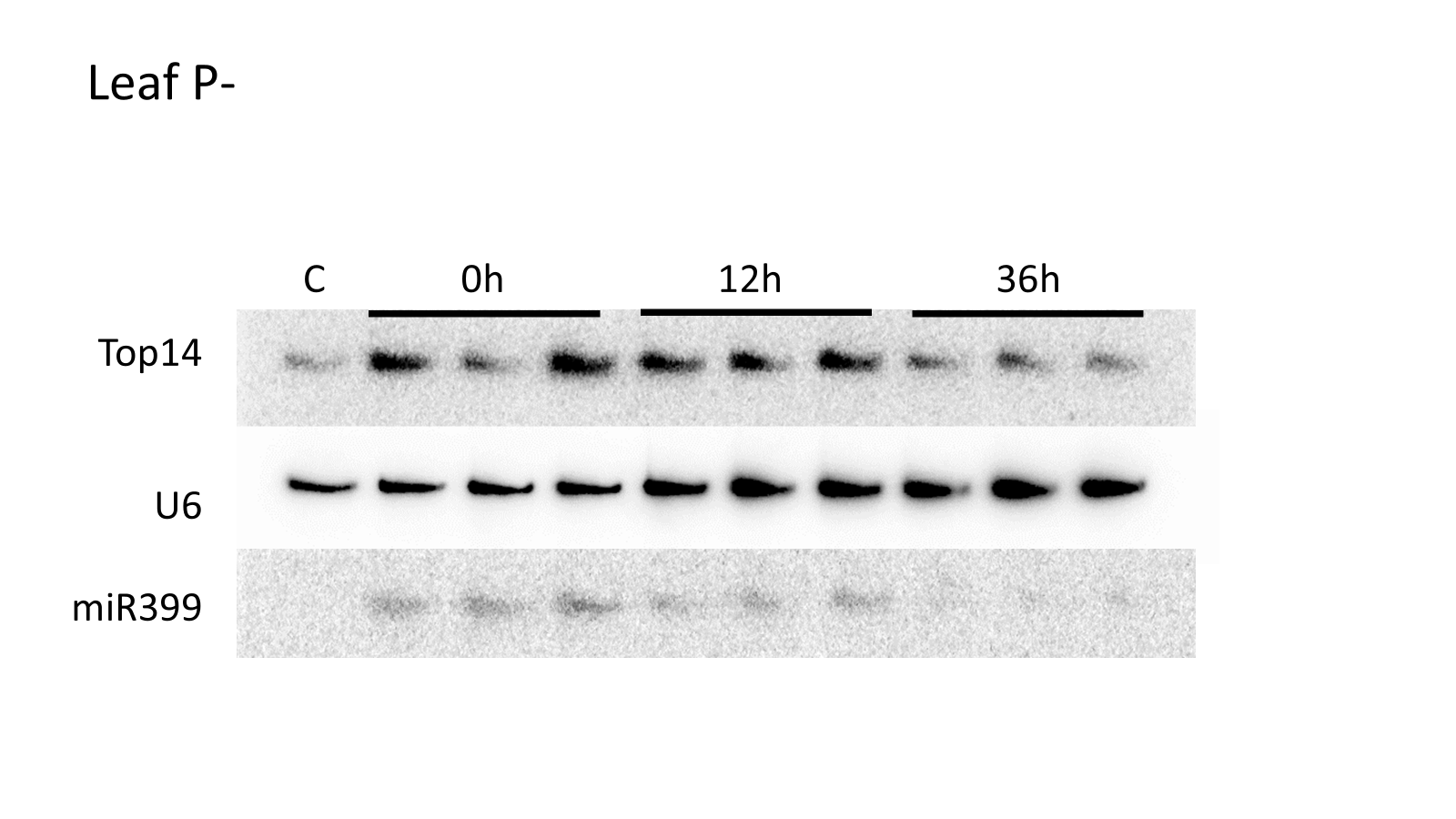

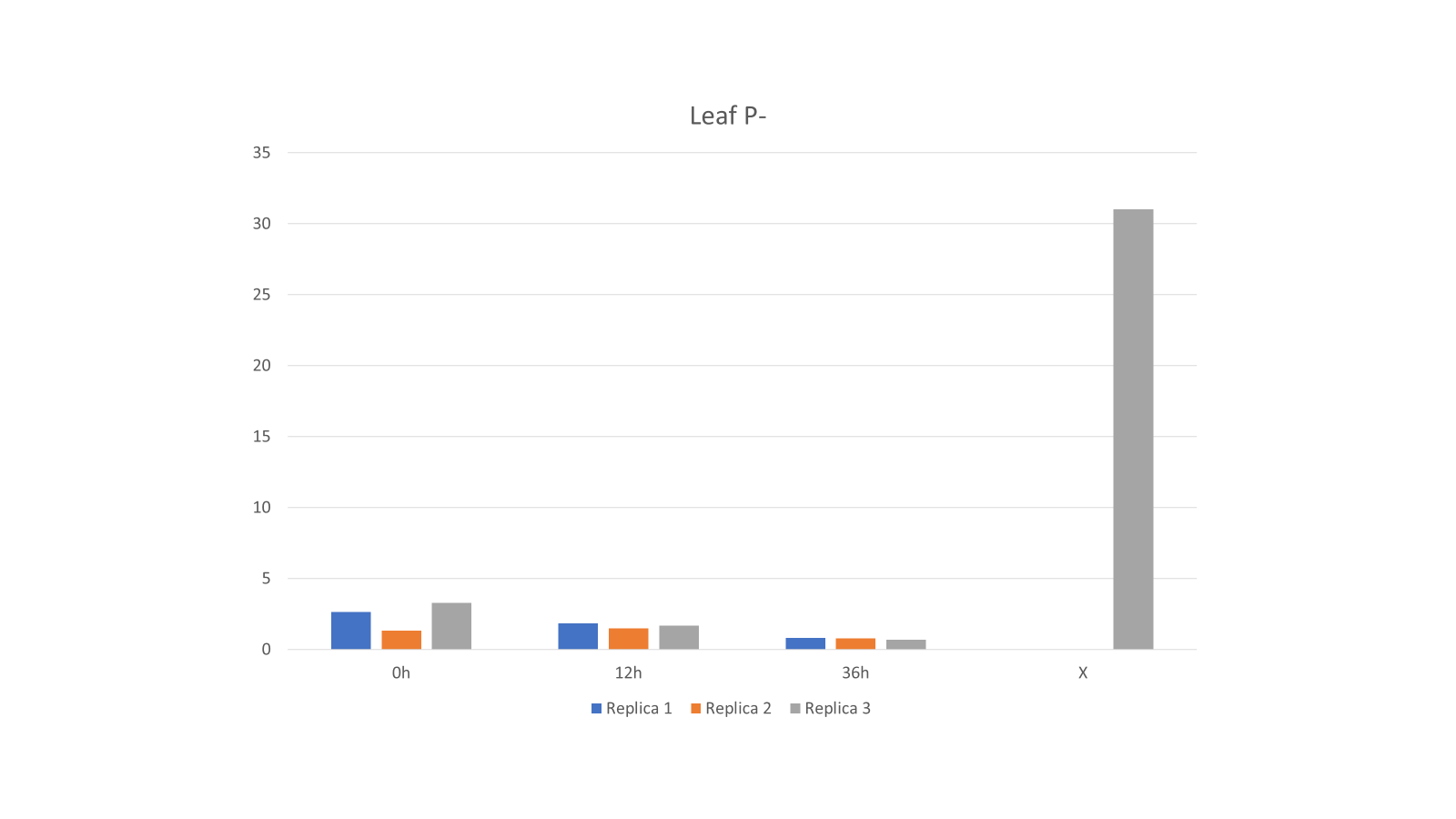


**Suppl. Fig. S2** One month old tomato plants were transferred to hydroponic culture in a modified Hogland’s nutrient solution (Van Delden et al. 2020). They were kept in full nutrient media for 3 days and at that point we changed the media of all of them to keep half of the plants with full nutrients (P+) and half in phosphate deprived media (P-). After six days in either P+ or P- conditions, we collected root, stem and leaf tissue from 6 plants in each condition (P+ 0h and P- 0h). The day after we added phosphate to the P- media and after 12h we collected again root, stem and leaf tissue from 6 plants in both groups (P+ 12h and P- 12h). Finally, 36h after replenishment we collected again root, stem and leaf tissue from 6 plants in both groups (P+ 36h and P- 36h). RNA was extracted from three biological replicates (different plants) per condition, resulting in a total of 54 samples (3 tissues x 3 timepoints x 3 replicates = 27 for both P+ and P-) and mature miRNA levels were assessed by northern blot. C was a loading control (diluted RNA from leaf P+ 36h), same in all 6 membranes to be able to compare them. U6 and miR399 were used as controls.

Van Delden SH, Nazarideljou MJ, Marcelis LFM (2020) Nutrient solutions for Arabidopsis thaliana: A study on nutrient solution composition in hydroponics systems. Plant Methods 16:1–14. https://doi.org/10.1186/S13007-020-00606-4/TABLES/4

| **Primer** | **Sequence 5’- 3’** |
| --- | --- |
| Sly pri-miRNA Fw | TGAAAGAATAATATAATGTACTGCATC |
| Sly pri-miRNA Rv | TAAAGTGTTACTTTATGTTGCATAGCT |
| Nbe pri-miRNA Fw | TGGTGACTTTGGTCCCGAAA |
| Nbe pri-miRNA Rv | GTTGGTGACTTTGGTCCCAAA |
| Pax pri-miRNA Fw | GTCAAATTTGCTACACTTGAAATGCC |
| Pax pri-miRNA Rv | GAGTTCTCAAACGTCTTTTTCTTTTT |
| Ini pri-miRNA Fw | CAAAAGTGAAGAAGATGGCAGTTTG |
| Ini pri-miRNA Rv | TCTCCAGCTGAACTAATCAAGCACT |

**Suppl. Table S1** Primers sequences used for pri-miRNA RT-PCR analyses in *Solanum lycopersicum* (Sly), *Nicotiana benthamiana* (Nbe), *Petunia axillaris* (Pax) and *Ipomoea nil* (Ini).

| **Primer** | **Sequence 5’- 3’** |
| --- | --- |
| Generacer RNA oligo | CGACUGGAGCACGAGGACACUGACAUGGACUGAAGGAGUAGAAA |
| Generacer oligo dT | GCTGTCAACGATACGCTACGTAACGGCATGACAGTG(T)_24_ |
| Generacer 5’ primer | CGACTGGAGCACGAGGACACTGA |
| Generacer 5’ nested | GGACACTGACATGGACTGAAGGAGTA |
| Generacer 3’ primer | GCTGTCAACGATACGCTACGTAACG |
| Sly T1 Rv | ATCCACAAGCGCAATCTCCACACAT |
| Sly T2 Rv | CGCAGTTAGCAGCAACAGGAGCAAG |
| Sly T3 Rv | GAATATTCAAGAAGCCCCGGCAACA |
| Sly T4 Rv | GGCCACAATATCCACGGCTTCCTTA |
| Sly T5 (LPR) Rv | ACTGTTGCTGTGTGCCGCGATGTAC |
| Sly T6 Rv | GCCCGCTTGACTGAGCTACCTGACT |
| Sly T8-9 Rv | GGACTGGAGGACACGGATGCTTCGA |
| Sly LA Rv | GCATTCAAGTAGAGCATATCCCTGTCAGGG |
| Sly SCW RV | GGCCCATTGCCCGCCATAACCGAT |
| Sly T1 Fw | ACACAAGTTCAGTTCCACCAGCAAGC |
| Sly T2 Fw | TGAGGGGTTGATGAATTTGGTCTCAAG |
| Sly T3 Fw | CGGGCTGCCGATAGTGCATTGTCTG |
| Sly T4 Fw | GTTTCCTTGGTGATGCAGGGCCAGG |
| Sly T5 (LPR) Fw | CCCCCACCCCACCCCTTTCTTCTTA |
| Sly T6 Fw | CTGATCCTGCAGCTCAGCTTGTGGC |
| Sly T8-9 Fw | CTCTTGCAAGCTGAACAGGCTGCCA |
| Sly T1 Rv nested | CTCCACACATCCAATGGCGA |
| Sly T2 Rv nested | GGAGCAAGTCCAGTAGTAAGTCC |
| Sly T3 Rv nested | GGCAACAATTTCGTGCGACT |
| Sly T4 Rv nested | ATTAGAAAGTACATCCCAAACCCCA |
| Sly T5 (LPR) Rv nested | TGGCATGTCTTGGAGTTCATCA |
| Sly T6 Rv nested | GAGATCAGTGCTGCCAGGAG |
| Sly T7 Rv nested | GCCGCAATTGACTGTACGAA |
| Sly T8-9 Rv nested | CCTTCGAAGCCCACTGGAAA |
| Sly C1 Rv | GCATTCAAGTAGAGCATATCCCTGTCAGGG |
| Sly C2 Rv | GGCCCATTGCCCGCCATAACCGAT |
| Nbe T1 Rv | CGCCACCAATCGACGACCCT |
| Nbe T2 Rv | GTGCAGTTGAAAGGCACTCAGCT |
| Nbe LPR 1&2 Rv | TGGTACATGCCAATCTTGAGTGAC |
| Nbe TCP4 Rv | TTCTGCATTACGTCGGTCCACTC |
| Nbe SCW-6 Rv | TGCCTGCCATAGCCGATATCAAA |
| Nbe T1 Fw | ACGAGACTGTGATTTCGCCGA |
| Nbe T2 Fw | TTGGTGATGCAGGGCCAGGATAT |
| Nbe LPR1 Fw | AGAGTGTTGGTGACTTTGTTCCT |
| Nbe LPR2 Fw | AAGGGTATTGGTGACTTTGGTCC |
| Nbe TCP4 Fw | GAATGGGAATGTTGCCAGTTCAA |
| Nbe Scarecrow-6 Fw | CTCCAGCTGCTTCCCCATTTT |
| Nbe T1 Rv nested | GGTTCCATTCTTCCCATCCCTCC |
| Nbe T2 Rv nested | TCAGTCTCCAAGCTCTTGTCGCA |
| Nbe LPR1 Rv nested | GTTTATCTTCTGCCATTGAAGCCAT |
| Nbe LPR2 Rv nested | TGGGCATGTCTGGAAGTTTGT |
| Nbe C1 Rv nested | CGTGGGTCAAAGAGCAGAAAATG |
| Nbe C2 Rv nested | TGGCAAATTGTGCAACTGGTG |
| Pax LPR Rv | GGCCTCGATTGTAGGTCCAGGGACTGT |
| Pax TCP4 Rv | TGCAGAAGGGAAGTTGCATTGGC |
| Pax SCW Rv | AGCCCATTGCCCGCCATAACCGA |
| Pax LPR Fw | AGGGTGTTTGTGACTTTGTCCTGCA |
| Pax TCP4 Fw | ACTCAGAAAGCAAAAGCCAAGCCCA |
| Pax SCW Fw | CAGAGCTGGTCCAGACGGGGAAT |
| Pax LPR Rv nested | CGAGTGACTTAGGGACATGAGCACCA |
| Pax C1 Rv nested | CCTGCCCCTCATCTGCACCTTCA |
| Pax C2 Rv nested | ACTGTGCAACTGGTGAGATCTCAGAGA |

**Suppl. Table S2** Primers sequences used for RLM-RACE analyses in *Solanum lycopersicum* (Sly), *Nicotiana benthamiana* (Nbe) and *Petunia axillaris* (Pax).

| **Species** | **miRNA sequence (3p)** | **miRNA* sequence (5p)** |
| --- | --- | --- |
| *Solanum lycopersicum* | CUUGGGACCAAAGUCACCAAC | UGGUGACUUUGAUCUCAAAAG |
| *Solanum pimpinellifolium* | CUUGGGACCAAAGUCACCAAC | UGGUGACUUUGAUCUCAAAAG |
| *Solanum arcanum* | CUUGGGACCAAAGUCACCAAC | UGGUGAUUUUGGUCUCAAAAG |
| *Solanum habrochaites* | CUUGGGACCAAAGUCACCAAC | UGGUGUCUUUGGUCUCAAAAG |
| *Solanum pennellii* | CUUGGGACCAAAGUCACCAAC | UGGUGACUUUGGUCUCAAAAG |
| *Solanum commersonii* | CUUGGGACCAAAGUCACCAAC | UGGUGACUUUGGUCUCAAAAG |
| *Solanum tuberosum* | CUUGGGACCAAAGUCACCAAC | UGGUGACUUUGGUCUCAAAAG |
| *Solanum melongena* | UUUGGGACCAAAGUCACCAAC | UGGUGCCUUUGGUCUCUAAAG |
| *Capsicum annuum* | CUUGGGACCAAAGUCACCAAC | UGGUGAUUUUGGUCUCAAAAG |
| *Nicotiana tabacum* | UUUGGGACCAAAGUCACCAAC | UGGUGACUUUGGUCUCGAAAG |
| *Nicotiana sylvestris* | UUUGGGACCAAAGUCACCAAC | UGGUGACUUUGGUCUCGAAAG |
| *Nicotiana benthamiana* | UUUGGGACCAAAGUCACCAAC | UGGUGACUUUGGUCCCGAAAG |
| *Nicotiana attenuata* | UUUGGGACCAAAGUCACCAAC | UGGUGACUUAUGGUCUCGAAAG |
| *Nicotiana otophora* | UUUGGAACCAAAGUCACCAAC | UGGUGACUUUGGUCUCGAAAG |
| *Nicotiana tomentosiformis* | UUUGGAACCAAAGUCACCAAC | UGGUGACUUUGGUCUCAAAAG |
| *Petunia axillaris* | UUUGGGACCAAAGUCACCAAC | UGGUGACUUUGGUCUCGAAAG |
| *Petunia integrifolia* | UUUGGGACCAAAGUCACCAAC | UGGUGACUUUGGUCUCGAAAG |
| *Ipomoea batatas* | UUUGGGACCAAAGUCACCAAC | UGGUGACUUUGUACCCAAAGG |
| *Ipomoea trifida* | UUUGGGACCAAAGUCACCAAC | UGGUGACUUUGUACCCAAAAC |
| *Ipomoea nil* | UUUGGGACCAAAGUCACCAAC | UGGUGACUUUGUAUCCAAAAC |

**Suppl. Table S3** miRNAtop14-5p and miRNAtop14-3p sequences in all the species in which the miRNA has been identified. The more abundant mature miRNAtop14 is located on the 3’ arm (3p) and the less abundant miRNAtop14* is on the 5’ arm (5p) of the miRNA hairpin. In red, nucleotides mismatching with *Solanum lycopersicum* sequence. National Center for Biotechnology Information (NCBI), SGN and Sweetpotato GARDEN databases were used to obtain these sequences (Fernandez-Pozo et al. 2015; Hirakawa et al. 2015; Sayers et al. 2022).

| **Species** | **A)**  **Distance miRNA-miRNA*** | **B)**  ***MIR* transcript predicted?** |
| --- | --- | --- |
| *Solanum lycopersicum* | 652 nt | yes, ncRNA |
| *Solanum pimpinellifolium* | 660 nt | no |
| *Solanum arcanum* | 619 nt | no |
| *Solanum habrochaites* | 640 nt | no |
| *Solanum pennellii* | 660 nt | no |
| *Solanum commersonii* | 669 nt | No |
| *Solanum tuberosum* | 668 nt | yes, EST |
| *Solanum melongena* | 872 nt | yes, EST |
| *Capsicum annuum* | 835 nt | No |
| *Nicotiana tabacum* | 932 nt | yes, ncRNA |
| *Nicotiana sylvestris* | 933 nt | yes, ncRNA |
| *Nicotiana benthamiana* | 899 nt | no |
| *Nicotiana attenuata* | 884 nt | yes, ncRNA |
| *Nicotiana otophora* | 1159 nt | yes, ncRNA |
| *Nicotiana tomentosiformis* | 1179 nt | no |
| *Petunia axillaris* | 48 nt | yes, TSA |
| *Petunia integrifolia* | 62 nt | yes, TSA |
| *Ipomoea batatas* | 491 nt | yes, TSA |
| *Ipomoea trifida* | 431 nt | no |
| *Ipomoea nil* | 441 nt | yes, ncRNA |

**Suppl. Table S4** miRNAtop14-miRNAtop14* distance and *MIRtop14* transcript prediction. **A** Distance in nucleotides between miRNAtop14 and miRNAtop14* in the genome of the species predicted to harbour *MIRtop14* (miRNA and miRNA* sequences not included). **B** Species in which *MIRtop14* has been predicted to be transcribed according to NCBI database (Sayers et al. 2022). ncRNA=non-coding RNA predicted, nucleotide collection. EST=transcript only detected in the EST collection. TSA=transcript only detected in the TSA collection

**Suppl. Table S5** *Nicotiana benthamiana* miRNAtop14 predicted targets by psRNAtarget server. **Column 1** Target accession and description, according to SGN transcript cDNA library Niben101. **Column 2** Expectation (Exp.), a score for miRNA-target complementarity, was set to a maximum of 2.5. **Column 3** UPE, target accessibility as the maximum energy to unpair the target site, was set to a maximum of 25. **Column 4** miRNA-target alignment gives the position of the first aligning nucleotide of the target and the last aligning nucleotide of the miRNA, considering the first position the 5’ end in both cases. The sequence of the target is written from the 5’ to the 3’ end (from left to right) and the sequence of the miRNA in the opposite direction. **Column 5** miRNA mode of action was predicted to be cleavage whenever there were not mismatches between target and miRNA nucleotides 9 and 11, and translational repression otherwise. Transcripts later analysed by RLM-RACE are shadowed in grey.

**Suppl. Table S6** Analysis of miRNAtop14-*LPR* complementarity in *Solanales* in which miRNAtop14 has been detected using psRNAtarget. **Column 1** *Solanales* species. **Column 2** Number of *LPR* paralogous found in the species analysed. **Column 3** Expectation (Exp.), a score for miRNA-target complementarity, was set to a maximum of 5, less possible restrictive value. **Column 4** UPE, target accessibility as the maximum energy to unpair the target site, was set to a maximum of 100, less possible restrictive value. **Column 5** miRNA-target alignment gives the position of the first aligning nucleotide of the target and the last aligning nucleotide of the miRNA, considering the first position the 5’ end in both cases. The sequence of the target is written from the 5’ to the 3’ end (from left to right) and the sequence of the miRNA in the opposite direction. **Column 6** miRNA mode of action was predicted to be cleavage whenever there were not mismatches between target and miRNA nucleotides 9 and 11, and translational repression otherwise. **Note,** in two species the analysis could not be carried out: in *Solanum pimpinellifolium*, in which *LPR* mRNA sequence was lacking the 5’UTR region, and in *Ipomoea batatas,* where not *LPR* sequence could be found.
